# GTPase Activity Plays a Key Role in the Pathobiology of LRRK2

**DOI:** 10.1371/journal.pgen.1000902

**Published:** 2010-04-08

**Authors:** Yulan Xiong, Candice E. Coombes, Austin Kilaru, Xiaojie Li, Aaron D. Gitler, William J. Bowers, Valina L. Dawson, Ted M. Dawson, Darren J. Moore

**Affiliations:** 1 NeuroRegeneration and Stem Cell Programs, Institute for Cell Engineering, Johns Hopkins University School of Medicine, Baltimore, Maryland, United States of America; 2 Departments of Neurology, Johns Hopkins University School of Medicine, Baltimore, Maryland, United States of America; 3 Department of Molecular Biology and Genetics, Johns Hopkins University School of Medicine, Baltimore, Maryland, United States of America; 4 Department of Cell and Developmental Biology, University of Pennsylvania, Philadelphia, Pennsylvania, United States of America; 5 Center for Neural Development and Disease, Department of Neurology, University of Rochester Medical Center, Rochester, New York, United States of America; 6 Department of Physiology, Johns Hopkins University School of Medicine, Baltimore, Maryland, United States of America; 7 Solomon H. Snyder Department of Neuroscience, Johns Hopkins University School of Medicine, Baltimore, Maryland, United States of America; 8 Brain Mind Institute, School of Life Sciences, École Polytechnique Fédérale de Lausanne, Lausanne, Switzerland; Massachusetts General Hospital, United States of America

## Abstract

Mutations in the *leucine-rich repeat kinase 2* (LRRK2) gene are associated with late-onset, autosomal-dominant, familial Parkinson's disease (PD) and also contribute to sporadic disease. The *LRRK2* gene encodes a large protein with multiple domains, including functional Roc GTPase and protein kinase domains. Mutations in LRRK2 most likely cause disease through a toxic gain-of-function mechanism. The expression of human LRRK2 variants in cultured primary neurons induces toxicity that is dependent on intact GTP binding or kinase activities. However, the mechanism(s) underlying LRRK2-induced neuronal toxicity is poorly understood, and the contribution of GTPase and/or kinase activity to LRRK2 pathobiology is not well defined. To explore the pathobiology of LRRK2, we have developed a model of LRRK2 cytotoxicity in the baker's yeast *Saccharomyces cerevisiae*. Protein domain analysis in this model reveals that expression of GTPase domain-containing fragments of human LRRK2 are toxic. LRRK2 toxicity in yeast can be modulated by altering GTPase activity and is closely associated with defects in endocytic vesicular trafficking and autophagy. These truncated LRRK2 variants induce similar toxicity in both yeast and primary neuronal models and cause similar vesicular defects in yeast as full-length LRRK2 causes in primary neurons. The toxicity induced by truncated LRRK2 variants in yeast acts through a mechanism distinct from toxicity induced by human α-synuclein. A genome-wide genetic screen identified modifiers of LRRK2-induced toxicity in yeast including components of vesicular trafficking pathways, which can also modulate the trafficking defects caused by expression of truncated LRRK2 variants. Our results provide insight into the basic pathobiology of LRRK2 and suggest that the GTPase domain may contribute to the toxicity of LRRK2. These findings may guide future therapeutic strategies aimed at attenuating LRRK2-mediated neurodegeneration.

## Author Summary

Parkinson's disease (PD) is the second most common neurodegenerative disorder. PD is considered to be caused by a combination of risk factors including environmental exposure, age, and a positive family history for disease. Several genes have been unambiguously implicated in PD. However, our knowledge is still limited about these genes and the disease mechanisms involved. Mutations in the *LRRK2* gene account for up to 40% of PD in certain populations. Since a single-cell model, baker's yeast, has been employed successfully to study the function of genes related to PD and other neurodegenerative disorders, we developed a yeast model of LRRK2 cytotoxicity in this study to investigate the function of LRRK2. We dissected the LRRK2 protein into different fragments including the various functional domains and found that fragments including the GTPase domain of LRRK2 are toxic. This toxicity can be modulated by alterations in GTPase activity and correlates with defects in cellular trafficking. These truncated LRRK2 variants induce similar toxicity and trafficking defects in both yeast and primary neuronal models. This yeast model reveals an important role of GTPase activity in the basic pathobiology of LRRK2 and may guide future therapeutic strategies for PD.

## Introduction

Parkinson's disease (PD (OMIM #168600)) is a common neurodegenerative movement disorder that is characterized by muscular rigidity, bradykinesia, resting tremor and postural instability [1],[2]. Although typically a sporadic disease, mutations in the *leucine-rich repeat kinase 2* (*LRRK2*, PARK8, OMIM #607060, GenBank #AY792511) gene have been identified as a cause of late-onset, autosomal dominant familial PD that is clinically and neurochemically indistinguishable from sporadic PD [3]–[7]. Importantly, *LRRK2* pathogenic mutations also contribute to sporadic PD [4],[8]. Mutations in *LRRK2* are the most common cause of familial and sporadic PD identified to date [9]. The *LRRK2* gene encodes a large protein of 2527 amino acids that contains multiple domains. These include a LRRK2-specific repeat region, multiple leucine-rich repeats, a *R*as *o*f *C*omplex (Roc) GTPase domain, a *C*-terminal *o*f *R*oc (COR) domain, and a protein kinase domain belonging to the tyrosine kinase-like protein kinase family [10],[11]. LRRK2 exhibits kinase activity whereby it can undergo autophosphorylation and can phosphorylate generic substrates [12]–[18]. However, physiological substrates for the kinase activity of LRRK2 have not yet been identified. The GTPase domain of LRRK2 can mediate GDP (guanosine-5′-diphosphate)/GTP (guanosine-5′-triphosphate) binding as well as GTP hydrolysis albeit at a relatively slow rate compared to other small GTPases such as Ras [14], [15], [19]–[22]. Intriguingly, GTP binding markedly enhances the kinase activity of LRRK2 and is an essential requirement for kinase activity [14],[15],[21],[22]. It is unclear at present how the GTP binding and GTP hydrolysis activities of LRRK2 are regulated. Disease-associated mutations located throughout the LRRK2 protein have been shown to variably alter GTP binding, GTP hydrolysis or kinase activity [14]–[24]. Thus, alterations in both GTPase and protein kinase activity are clearly important for the development of PD due to LRRK2 mutations.

A number of useful models have been developed to investigate the pathobiology of LRRK2 disease-associated variants, including *Drosophila*, transgenic mice and primary neuronal models. Studies in cultured primary cortical neurons reveal that the exogenous expression of pathogenic mutant forms of full-length human LRRK2 (i.e. G2019S, R1441C and Y1699C) induces marked neuronal toxicity relative to the wild-type protein [21]–[23]. Wild-type LRRK2 can also induce neuronal toxicity but to a lesser degree. LRRK2-induced toxicity in this neuronal model is dependent on intact GTP binding and kinase activity [21]–[23]. In *Drosophila* models, expression of human LRRK2 variants induces selective dopaminergic neurodegeneration and motor dysfunction [25]–[27]. Mutant LRRK2 R1441G BAC transgenic and R1441C knock-in mice exhibit mild defects in dopaminergic neurotransmission and motor deficits [28],[29]. These observations are consistent with a toxic gain-of-function mechanism for disease-associated LRRK2 variants. The molecular mechanism(s) and/or pathway(s) by which LRRK2 variants induce neuronal toxicity are poorly understood and how alterations in GTPase or kinase activities regulate the toxic effects of LRRK2 are not well defined.

Model organisms including yeast, worms, flies and mice are commonly used to uncover the fundamental biology and pathobiology of proteins associated with neurodegenerative diseases, including poly-glutamine expansion disorders, Parkinson's disease, Alzheimer's disease, Prion diseases and Friedreich's ataxia. The baker's yeast *Saccharomyces cerevisiae*, a eukaryotic single-cell organism, provides a powerful experimental system in which to dissect complex biological pathways and processes. Major advantages of yeast include the high degree of conservation of pathways, processes and protein function with mammalian cells, and the accessibility of yeast cells to genetic manipulation and genome-wide screening approaches. For Parkinson's disease (PD), yeast have provided unique insight into the basic biology and pathobiology of the α-synuclein protein that is associated with autosomal dominant familial PD [30]–[33]. Here, we have employed yeast as a model to further understand the basic pathobiology of LRRK2. Expression of truncated human LRRK2 reduces yeast viability in a manner largely dependent on the GTPase domain of this protein. Reduced viability in this yeast LRRK2 model is independent of kinase activity and disease-associated mutations, but can be modulated instead by altering GTPase activity and is associated with defects in vesicular trafficking and autophagy. This yeast model provides insight into the basic pathobiology of LRRK2 and suggests that the GTPase domain may contribute to the cellular toxicity of LRRK2. These findings may guide future therapeutic strategies aimed at attenuating LRRK2-mediated neurodegeneration.

## Results

### Expression of Human LRRK2 Domain Fragments Reduces Yeast Viability

To gain novel insight into the pathobiology of LRRK2, we set out to develop a simple yeast LRRK2 model. Yeast cells were transformed with expression constructs that express at high copy V5-tagged full-length human LRRK2 under the control of the galactose-inducible *GAL1* promoter. Expression of wild-type (WT) or G2019S LRRK2 variants fail to affect the viability of yeast cells, which is most likely due to the formation of large LRRK2-positive intracytoplasmic inclusions that are biochemically insoluble (Figure S1). The same results are observed with low copy expression constructs (Figure S1). Thus, we elected to examine the detrimental effects of various smaller protein fragments of human LRRK2 that contain different functional domains.

**Figure 1:**
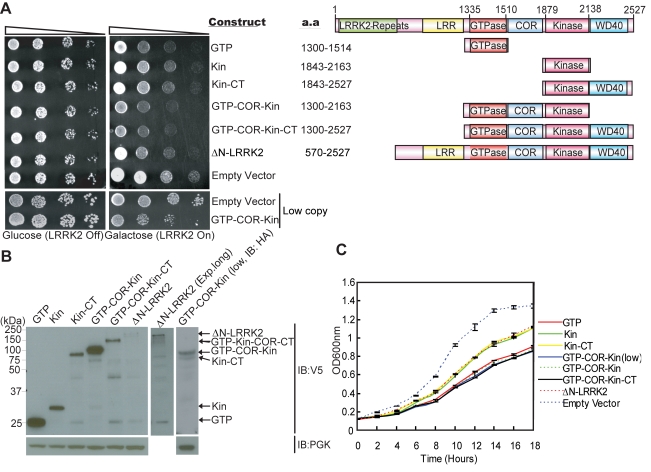
Expression of LRRK2 domain fragments reduces the viability of yeast. (A) LRRK2 domain fragments reduce yeast viability. Yeast cells (BY4741 MATa) were transformed with galactose-inducible high copy expression constructs containing the following human LRRK2 domain fragments: GTPase domain (GTP, residues 1300–1514), kinase domain (Kin, residues 1843–2163), kinase domain plus the C-terminal region (Kin-CT, residues 1843–2527), GTPase-COR-kinase domains (GTP-COR-Kin, residues 1300–2163), GTPase-COR-kinase domains plus the C-terminal region (GTP-COR-Kin-CT, residues 1300–2527), and a LRRK2 fragment lacking the N-terminal LRRK2-specific repeat region (ΔN-LRRK2, residues 570–2527) and low copy expression construct containing the GTP-COR-Kin region. Empty vectors (pYES/CT, p416GAL) are used as controls. Cells were spotted onto media containing glucose (LRRK2 Off, repressed, left panel) or galactose (LRRK2 On, induced, right panel) and incubated at 30°C for 2–3 days. Shown are five-fold serial dilutions (from left to right, as indicated by graded open box) starting with equal numbers of cells. Protein domain structure of each LRRK2 fragment relative to the full-length protein is also indicated. (B) Expression of LRRK2 domain fragments in yeast cells following galactose induction was detected by Western blot analysis with anti-V5 antibody, with anti-PGK antibody as a protein loading control. (C) Growth curve analysis in liquid media containing galactose was used to monitor the growth rate of yeast cells expressing each LRRK2 domain fragment or with empty vector as a control. Data are taken from three independent experiments with each data point representing the mean ± SEM (*n* = 3).

Following galactose induction of high copy expression constructs, LRRK2 fragments minimally containing the GTPase domain markedly reduce yeast viability relative to control cells, with the most toxic fragment containing the central GTPase, COR and kinase domains (GTP-COR-Kin) of LRRK2 (Figure 1A). A larger LRRK2 fragment additionally containing the C-terminus (GTP-COR-Kin-CT) reduces yeast viability to a similar extent. The GTPase domain alone is also sufficient to markedly reduce yeast viability (Figure 1A). LRRK2 fragments containing the kinase domain alone (Kin or Kin-CT) or a fragment lacking the N-terminal region (ΔN-LRRK2), which is poorly expressed, are much less toxic to yeast (Figure 1A). Western blot analysis confirms the expression of each LRRK2 fragment in yeast following galactose induction (Figure 1B). LRRK2 fragments exhibit similar diffuse cytoplasmic localization patterns in yeast as revealed by fluorescence microscopy (Figure S2). The loss of viability due to the expression of each LRRK2 fragment is confirmed by monitoring the growth rate of yeast cells in liquid media following galactose induction (Figure 1C).

We focused further on the GTP-COR-Kin fragment of LRRK2 throughout this study since its expression is most toxic to yeast cells and because it permits further analysis of the contribution of both enzymatic domains. To test if the toxicity is dose-dependent, we also examined the effects of low copy expression of the GTP-COR-Kin fragment. A similar phenotype is observed as with high copy expression of the GTP-COR-Kin fragment (Figure 1A and 1C). Thus, the protein length, expression levels or cellular localization of each LRRK2 fragment do not correlate with their effects on yeast viability suggesting that alterations in viability are dependent on the protein domain composition or activity of each LRRK2 fragment. Moreover, these data demonstrate that LRRK2 protein fragments that contain the GTPase domain, but not full-length LRRK2, can reduce the viability of yeast cells.

### GTPase Activity Modulates LRRK2-Induced Toxicity in Yeast

**Figure 2:**
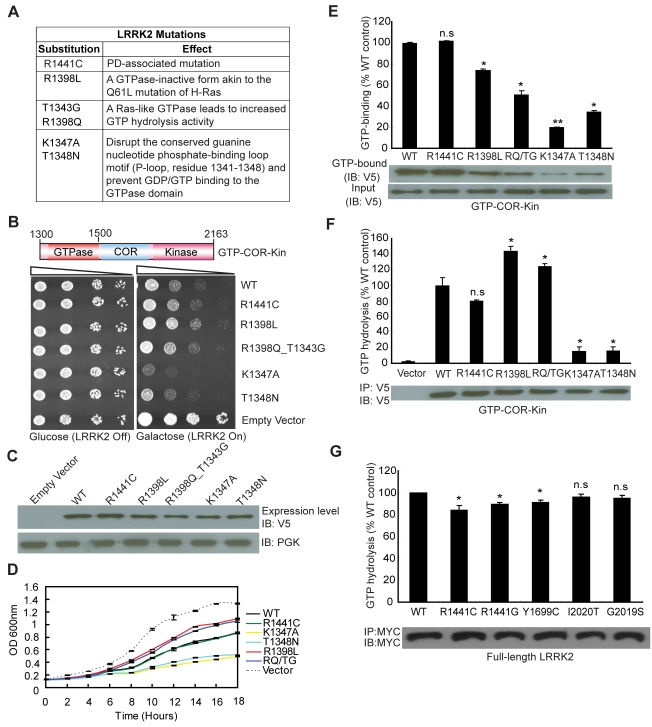
GTPase activity modulates LRRK2-induced toxicity in yeast. (A) Table of LRRK2 sequence variants employed in this study and their predicted functional effects. (B) GTPase mutations, K1347A and T1348N, markedly enhance LRRK2-induced toxicity in yeast compared to WT or other GTPase mutations. Yeast cells were transformed with galactose-inducible expression constructs containing the central GTP-COR-Kin fragment of LRRK2 harboring various functional GTPase variants (WT, R1441C, R1398L, R1398Q/T1343G, K1347A, and T1348N) or empty vector as a control. Spotting experiments were conducted to examine the viability of yeast cells due to the expression of each truncated LRRK2 GTPase variant. Shown are five-fold serial dilutions (from left to right, as indicated by graded open box) starting with equal numbers of cells grown on media containing glucose (LRRK2 Off, left panel) or galactose (LRRK2 On, right panel). (C) Expression of LRRK2 GTPase variants in the GTP-COR-Kin fragment in yeast following galactose induction was detected by Western blot analysis with anti-V5 antibody, with anti-PGK antibody as control for protein loading. (D) Growth curve analysis in liquid media containing galactose was used to measure the growth rate of yeast cells expressing each truncated LRRK2 GTPase variant relative to an empty vector control. Data are taken from three independent experiments with each data point representing the mean ± SEM (*n* = 3). (E) GTP-binding activity was determined for each LRRK2 GTPase variant (in the GTP-COR-Kin fragment) derived from yeast cell lysates following galactose induction by measuring the relative levels of GTP-bound LRRK2 with normalization to input levels of total LRRK2. LRRK2 levels were determined from western blot images by densitometric analysis. Data are expressed as GTP-binding as a percent of WT LRRK2 levels with each bar representing the mean ± SEM from three independent experiments. An example Western blot probed with anti-V5 antibody is shown indicating the levels of GTP-bound GTP-COR-Kin LRRK2 fragment and input levels. (F) GTP hydrolysis activity was determined in yeast by measuring the concentration of free P_i_ released from GTP for each truncated LRRK2 GTPase variant and normalized to LRRK2 input levels. Input levels of immunoprecipitated GTP-COR-Kin LRRK2 derived from yeast total lysates were detected by Western blot analysis with anti-V5 antibody, as shown, with densitometric analysis. GTP hydrolysis activity for each LRRK2 variant is expressed as P_i_ release as a percent of WT LRRK2 activity with each bar representing the mean ± SEM from three independent experiments. (G) GTP hydrolysis activity was measured for disease-associated mutations in full-length human LRRK2. Input levels of immunoprecipitated myc-tagged LRRK2 derived from HEK-293T cell lysates were detected by Western blot analysis with anti-MYC antibody, as shown, with densitometric analysis. GTP hydrolysis activity for each LRRK2 variant is expressed as P_i_ release as a percent of WT LRRK2 activity with each bar representing the mean ± SEM from five independent experiments. Data were analyzed for statistical significance by two-tailed unpaired Student's *t*-test compared to WT-LRRK2 (*P<0.01 and **P<0.001). *n.s.*, non-significant.

Since expression of the GTPase domain of LRRK2 is sufficient to markedly reduce yeast viability, we sought to determine whether alterations in GTPase activity could influence this growth deficit. A number of missense mutations were introduced into the GTPase domain within the GTP-COR-Kin LRRK2 fragment that are predicted to functionally alter enzymatic activity (Figure 2A). Two mutations, K1347A and T1348N, disrupt the conserved guanine nucleotide phosphate-binding loop motif (P-loop, residues 1341–1348) and prevent GDP/GTP binding to the GTPase domain [15],[22]. Two other mutations, R1398L and R1398Q, were targeted at the R1398 residue, a highly conserved glutamine residue in most small GTPases (i.e. Q61 in H-Ras). LRRK2 contains a highly conserved DFAGR motif (residues 1394–1398) in the switch II region which is mainly responsible for GTP hydrolysis. The P-loop residue T1343 is a glycine residue (G12) in H-Ras. In H-Ras, the combined G12V and Q61L mutations create a GTPase-inactive form of this protein, which is constitutively GTP-bound and active. We introduced these two key H-Ras residues into LRRK2 via the analogous mutations T1343G and R1398Q (RQ/TG) to create a Ras-like GTPase that leads to increased GTP hydrolysis activity (Figure 2A) [15]. Moreover, a common R1441C pathogenic variant was also introduced into the GTPase domain of LRRK2. Expression of the GTP-COR-Kin fragment of LRRK2 containing each mutation was induced by spotting yeast cells onto galactose media. Remarkably, altering the GTPase activity of LRRK2 leads to marked changes in yeast viability (Figure 2B). Compared to WT LRRK2, the GTP binding-deficient mutants K1347A and T1348N cause a dramatic reduction in yeast viability whereas the mutant R1398L and Ras-like mutant RQ/TG partially improve viability (Figure 2B). The disease-associated R1441C variant reduces yeast viability similar to WT LRRK2 (Figure 2B). Western blot analysis reveals that each mutant LRRK2 fragment is expressed at similar levels, which excludes alterations in expression level as a cause of their differential effects on yeast viability (Figure 2C). Furthermore, fluorescence microscopic analysis fails to reveal obvious differences in the cellular localization of truncated LRRK2 GTPase variants with each variant adopting a similar diffuse cytoplasmic distribution in yeast cells (Figure S2). Growth impairments induced by expression of each mutant LRRK2 fragment in yeast are further confirmed in liquid media following galactose induction (Figure 2D).

To determine how alterations in the GTPase activity of LRRK2 due to each functional mutation correlate with changes in yeast viability, we examined both the GTP binding and GTP hydrolysis activities of each mutant LRRK2 fragment. GTP binding was measured using an established GTP-sepharose pull-down assay on total yeast proteins expressing each LRRK2 fragment (Figure 2E). WT and the disease-associated mutant R1441C LRRK2 bind to immobilized GTP to similar extents whereas surprisingly all other mutants exhibit significantly reduced GTP binding (Figure 2E). Consistent with prior reports of full-length LRRK2 [14], [15], [19]–[22], the P-loop mutations, T1348N and K1347A, impair the GTP binding of LRRK2 (Figure 2E). Importantly, the GTP binding capacity of each LRRK2 GTPase mutant does not correlate with its effects on yeast viability. It is not currently possible to measure the capacity of each mutant LRRK2 fragment to bind GDP. It is likely that certain mutations (i.e. the P-loop mutants K1347A and T1348N) impair GDP/GTP binding whereas other mutations (i.e. the Ras-like mutant RQ/TG and R1398L) may alter the affinity for binding to GDP and GTP.

The effects of each mutation on LRRK2-mediated GTP hydrolysis were also determined *in vitro* by measuring the release of the γ-phosphate moiety from GTP (Figure 2F). Truncated WT LRRK2 displays detectable GTP hydrolysis activity whereas the R1441C mutant exhibits a small reduction in activity, similar to previous reports [14],[19],[20]. As expected, the Ras-like RQ/TG mutant leads to a marked increase in GTP hydrolysis activity but unexpectedly the R1398L mutant produces a similar increase in activity. The P-loop mutants K1347A and T1348N essentially abolish the GTP hydrolysis activity of LRRK2 as expected (Figure 2F). Therefore, alterations in GTP hydrolysis activity of each truncated LRRK2 GTPase mutant correlate closely with their effects on yeast viability. In this case, increased GTP hydrolysis partially improves the viability of yeast compared to WT LRRK2 whereas impaired hydrolysis dramatically reduces yeast viability. Notably, alterations in kinase activity via introduction of kinase-impaired (i.e. K1906M or T2031A/S2032A/T3035A) or a kinase-hyperactive (i.e. G2019S) mutation fails to similarly influence LRRK2-induced toxicity in yeast (Figure S3).

To further examine if GTPase activity plays a key role in the toxic process, we investigated the GTPase activity of full-length human LRRK2 harboring the most frequent mutations causing PD. Importantly, the mutations R1441C/G in the GTPase domain and Y1699C in the adjacent COR domain, significantly decrease GTPase activity (Figure 2G) although the mutations, G2019S and I2020T, in the kinase domain do not have a significant effect, suggesting that impaired GTP hydrolysis of LRRK2 can contribute to PD.

### Expression of LRRK2 Causes Defects in Endocytic Vesicular Trafficking and Autophagy

In yeast cells expressing human α-synuclein (SNCA, PARK1/4, OMIM #163890, GenBank #BC108275), defects in vesicular trafficking have been shown to underlie the cytotoxic effects of this protein with the earliest defect being a block in ER-to-Golgi vesicular trafficking [30]–[32]. Since α-synuclein pathology is a common feature of patients with LRRK2 mutations [3],[7],[34], vesicular trafficking was examined to determine whether similar defects could also underlie LRRK2-induced toxicity in yeast. The lipophilic fluorescent dye, FM4–64, is useful for monitoring endocytosis in yeast. FM4–64 binds to the plasma membrane of yeast cells where it is internalized by endocytosis into vesicles that subsequently undergo trafficking to the vacuole via the early and late endosome compartments. Thus, FM4–64 dye selectively stains the yeast vacuolar membrane appearing as a large ring-like cytoplasmic structure.

**Figure 3:**
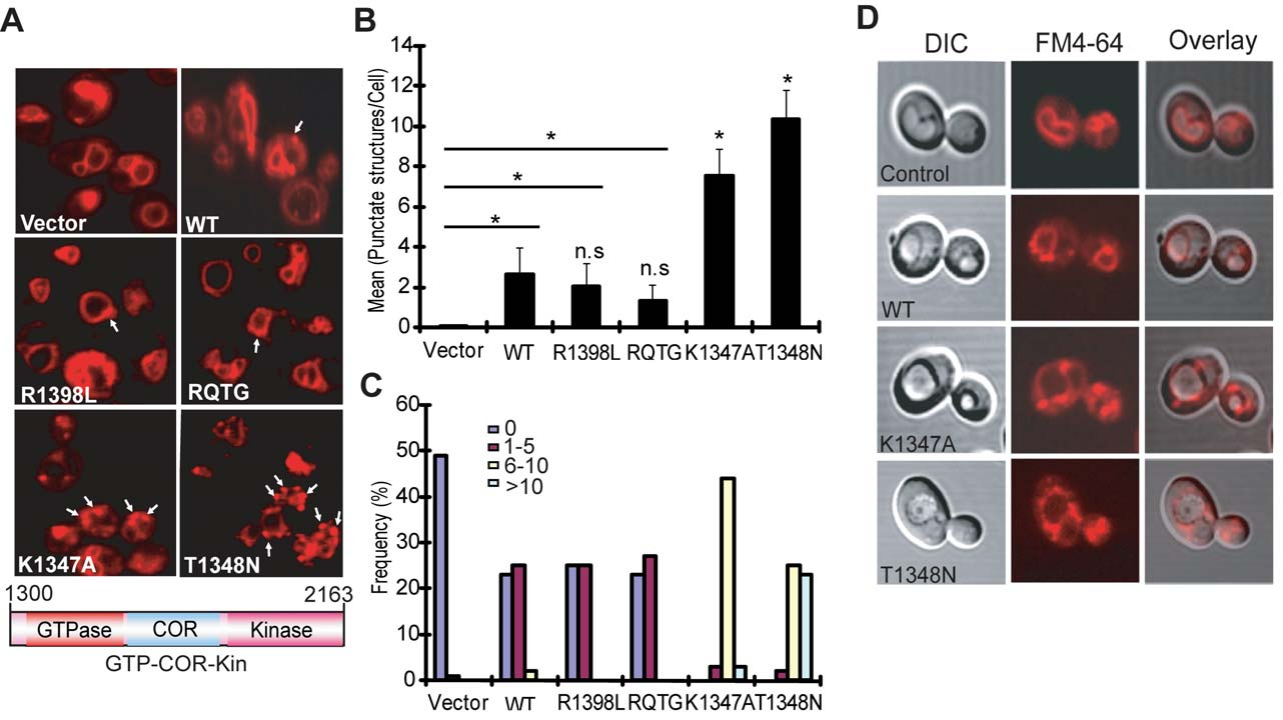
LRRK2 GTPase variants induce defects in endocytic vesicular trafficking. (A) Endocytosis of the lipophilic fluorescent dye FM4–64 (red) was employed to monitor the effects of LRRK2 GTPase variants (in the GTP-COR-Kin fragment) on vesicular trafficking to the vacuole in yeast. Cells carrying empty vector display normal ring-like vacuolar membrane staining (asterix). Expression of the GTPase mutants, K1347A or T1348N, markedly disrupts FM4–64 vacuole localization with the appearance of multiple large punctate structures (arrows). (B) Quantification of endocytic trafficking defect showing the average number of FM4–64-positive punctate structures per cell. A total of 100 cells were analyzed in one experiment and data are representative of at least two independent experiments. Bars represent the mean ± SEM. Data were analyzed for statistical significance by two-tailed unpaired Student's *t*-test relative to WT LRRK2, or by pair-wise comparisons with vector controls where indicated by horizontal lines (*P<0.01). *n.s.*, non-significant versus WT. (C) Frequency distribution showing the percent (%) of cells with different numbers of FM4–64-positive punctate structures as a measure of endocytic trafficking defects between truncated LRRK2 GTPase variants. Data are taken from one experiment (*n* = 100 cells) and are representative of at least two independent experiments. (D) Yeast cells carrying LRRK2 GTPase variants have normal vacuolar morphology. DIC images are employed to visualize the morphology of vacuoles for each LRRK2 construct following galactose induction and FM4–64 staining.

Yeast cells expressing truncated LRRK2 variants following galactose induction were incubated with FM4–64 dye and live-cell imaging was conducted by confocal fluorescence microscopy. WT LRRK2 expression partly disrupts the normal trafficking of FM4–64 to the vacuolar membrane relative to control cells, which exhibit normal ring-like vacuolar staining (Figure 3A). WT LRRK2 expression results in the appearance of large cytoplasmic punctate structures in addition to normal vacuolar staining, suggesting a modest defect in trafficking of FM4–64-labeled vesicles to the vacuole leading to their accumulation in endosomes. Yeast cells expressing truncated LRRK2 containing the two most toxic GTPase mutations, K1347A and T1348N, which impair the GTP binding and hydrolysis activity of LRRK2, exhibit severe defects in the endocytic trafficking pathway with a dramatic increase in the appearance of labeled punctate structures and the complete absence of normal vacuolar membrane staining (Figure 3A). Truncated LRRK2 variants that partially improved yeast viability compared to WT protein (i.e. RQ/TG and R1398L) induce similar trafficking defects to WT LRRK2 (Figure 3A). Normal FM4–64 labeling of vacuolar membranes is observed when yeast cells are grown in glucose media (data not shown). DIC images show that cells expressing each of the LRRK2 fragments have normal vacuolar morphology (Figure 3D).

Quantitation of defective endocytic trafficking reveals that the toxic GTPase-inactive mutants, K1347A and T1348N, lead to a significant increase in the number and frequency of FM4–64-labeled punctate structures per cell compared to WT LRRK2, whereas the GTPase-active mutants, RQ/TG and R1398L, display a small non-significant reduction in the number of punctate structures relative to WT (Figure 3B and 3C). Punctate structures are not normally observed in control yeast cells (Figure 3B and 3C). The vesicular trafficking defects induced by expression of each truncated LRRK2 GTPase variant in yeast do not correlate with alterations in their cellular localization (Figure S2). In particular, there is no specific enrichment in the vacuole or endosomal compartments of each LRRK2 variant that would obviously account for their differential effects on endocytic vesicular trafficking (Figure S2). These results indicate that the endocytic vesicular trafficking defect in yeast is associated with alterations in LRRK2 GTPase activity and likely underlies toxicity in yeast induced by truncated LRRK2.

**Figure 4:**
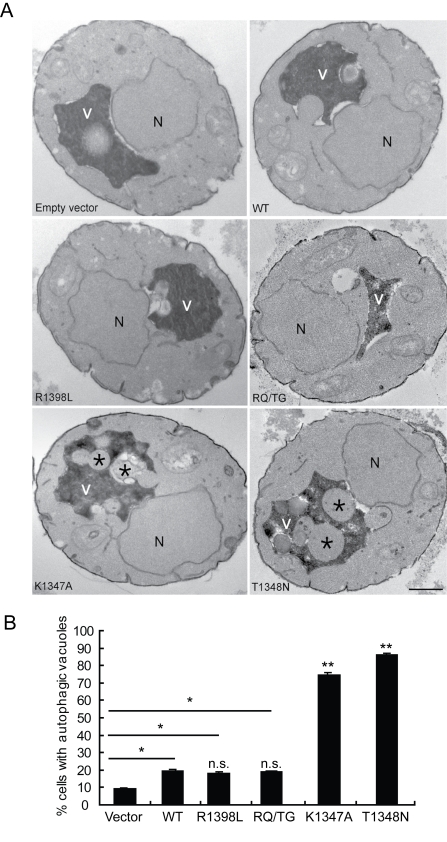
LRRK2 GTPase variants induce defects in autophagy. (A) Transmission electron micrographs demonstrating increased autophagic vacuoles (asterix) within the vacuole of yeast cells expressing the toxic GTPase variants, K1347A or T1348N, in the LRRK2 GTP-COR-Kin fragment. V, vacuoles; N, nucleus; Scale bars, 2 µm. (B) Quantification of autophagic defects showing the percentage of cells with autophagic vacuoles. A total of 100 cells were analyzed in one experiment and data are representative of at least two independent experiments. Bars represent the mean ± SEM. Data were analyzed for statistical significance by two-tailed unpaired Student's *t*-test relative to WT LRRK2, or by pair-wise comparisons with vector controls where indicated by horizontal lines (*P<0.01 and **P<0.001). *n.s.*, non-significant versus WT.

To verify that the observed defects induced by LRRK2 expression in yeast are due to vesicular trafficking pathways rather than simply by protein aggregation, yeast cells expressing truncated LRRK2 variants following galactose induction were examined by transmission electron microscopy (TEM) (Figure 4). Interestingly, yeast cells expressing truncated LRRK2 containing the two most toxic GTPase mutations, K1347A and T1348N, which impair GTPase activity exhibit a significant increase of autophagic vacuoles (AVs) (74.7% in K1347A cells and 86.2% in T1348N cells) compared to WT LRRK2 (19.4% AVs) (Figure 4A and 4B). In contrast, AVs were uncommon in yeast cells carrying empty vector (9.2% AVs) (Figure 4A and 4B). In accordance with fluorescence localization studies of truncated LRRK2 variants in yeast (Figure S2), protein aggregates or inclusions were not readily observed in the electron micrographs. Taken together these data indicate that LRRK2-induced trafficking defects are mediated at least in part by alterations in autophagy in addition to effects on the endocytic vesicular trafficking pathway.

To provide insight into the mechanism of LRRK2-induced toxicity in yeast, and to determine whether there are differences or similarities with α-synuclein-induced toxicity, a small candidate genetic screen was performed in yeast focused on modifiers of α-synuclein-induced toxicity. We elected to analyze potent modifiers of human α-synuclein-induced toxicity Ypt1 (GenBank #AAS56793) and Ykt6 (GenBank #AAB32050) [30],[31], as well as Hsp31 (Genbank #AAB64972), the yeast ortholog of human DJ-1 [33], a neuroprotective redox-responsive protein associated with familial PD (PARK7, OMIM #606324) [35],[36]. Yeast cells were transformed with constructs expressing truncated WT LRRK2, each candidate protein alone, or both proteins together under the control of the *GAL1* promoter and viability was examined by spotting of yeast cells on to galactose media. Expression of WT LRRK2 alone reduces yeast viability, whereas co-expression with each of the three candidate proteins fails to suppress the LRRK2-induced growth deficit (Figure S4). The three candidate yeast proteins were also tested for their ability to suppress toxicity due to the expression of the truncated LRRK2 variants, K1347A and T1348N, which induce a more pronounced loss of viability in yeast than WT LRRK2. Co-expression with each of the three candidate proteins also fails to suppress the K1347A- or T1348N-induced growth deficit (Figure S4). Collectively, our data demonstrate that known potent suppressors of α-synuclein-induced toxicity in yeast (i.e. Ypt1 and Ykt6) do not specifically suppress LRRK2-induced toxicity in this model suggesting that α-synuclein and LRRK2 induce toxicity in yeast through distinct pathways.

Following expression of truncated LRRK2 variants, we also fail to observe defects in the normal trafficking of carboxypeptidase Y (CPY) and alkaline phosphatase (ALP) proteins from the endoplasmic reticulum (ER) to the vacuole by pulse-chase analysis (data not shown), which represent two distinct biosynthetic transport pathways that converge upon the vacuole in addition to the endocytic pathway. Notably, human α-synuclein expression in yeast manifests prominent defects in normal CPY and ALP trafficking consistent with derangements in ER-to-Golgi vesicular trafficking [30]. Accordingly, toxicity induced by LRRK2 and α-synuclein expression in yeast most likely occur via impairment of distinct vesicular trafficking pathways.

### GTPase Activity Modulates LRRK2-Induced Neuronal Toxicity

**Figure 5:**
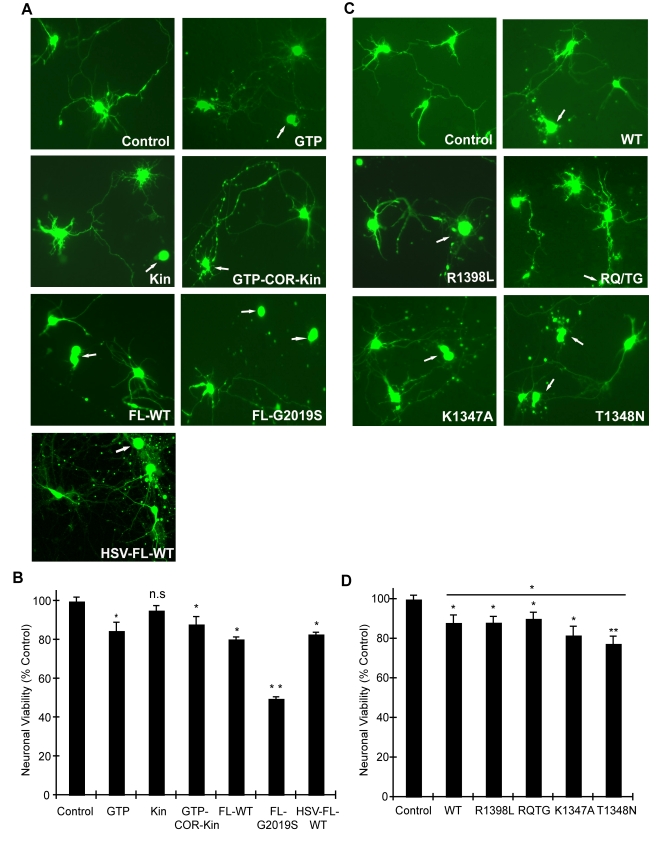
GTPase activity modulates LRRK2-induced neuronal toxicity. (A) Human LRRK2 domain fragments containing the GTPase domain (GTP and GTP-COR-Kin) but not the kinase domain (Kin) alone induce neuronal toxicity similar to full-length WT LRRK2. Representative fluorescent images (eGFP) showing mouse primary cortical neurons co-transfected with LRRK2 constructs and eGFP in a 10∶1 molar ratio or transduced with HSV-WT-LRRK2/CMV-eGFP virus expressing full-length WT LRRK2. Neuronal viability was analyzed at 48 hrs post-transfection (DIV 12) with non-viable neurons exhibiting obvious neurite process and/or nuclear fragmentation (arrows). (B) Quantification of neuronal viability induced by LRRK2 expression. Bars indicate the viability of eGFP-positive neurons (*n* = 200) for each transfection condition expressed as a percent (%) of control neurons (eGFP only). Data represent the mean ± SEM from three independent experiments. Data were analyzed for statistical significance by two-tailed unpaired Student's *t*-test compared to control neurons (*P<0.01 and **P<0.001). *n.s.*, non-significant. (C) LRRK2 GTPase variants (in GTP-COR-Kin fragment) induce neuronal toxicity. Representative fluorescent images (eGFP) of neurons at 48 hrs post-transfection containing truncated LRRK2 GTPase variants and eGFP. Arrows indicate non-viable neurons. (D) Quantification of neuronal viability induced by truncated LRRK2 GTPase variants. Bars indicate the viability of eGFP–positive neurons (*n* = 200) for each transfection condition expressed as a percent (%) of control neurons (eGFP only). Data represent the mean ± SEM from three independent experiments. Data were analyzed for statistical significance by two-tailed unpaired Student's *t*-test compared to control neurons, or by pair-wise comparisons where indicated by horizontal lines (*P<0.05 and **P<0.005). *n.s.*, non-significant versus control.

In order to validate the observations from this yeast model of LRRK2 toxicity and determine its wider applicability to mammalian cells, we examined the effects of human LRRK2 domain fragments and GTPase variants on neuronal viability. Expression constructs containing LRRK2 fragments identical to those employed in yeast including the GTPase domain (GTP), kinase domain (Kin) and the GTP-COR-Kin fragment as well as full-length WT or G2019S LRRK2 were individually co-transfected together with eGFP as a marker into mouse primary cortical neurons and their effects on neuronal viability were compared. A well-established assay was employed to examine the viability of eGFP-positive neurons containing LRRK2 based on neurite process length and fragmentation as a reliable indicator of neuronal viability [21],[22],[37],[38]. Using this method, LRRK2 expression was confirmed in >95% of eGFP-positive cortical neurons that were also positive for the neuronal marker, MAP2 (representative images in Figure S5A and S5C), and neuronal viability was also confirmed by TUNEL staining (representative images in Figure S5B). Expression of the GTPase domain, the GTP-COR-Kin fragment and full-length WT LRRK2 induces significant and equivalent neuronal toxicity relative to control neurons expressing eGFP alone, with a 10–20% loss of viability (Figure 5A and 5B). The kinase domain alone fails to significantly reduce neuronal viability. Full-length LRRK2 containing the common G2019S pathogenic variant serves as a positive control for toxicity and induces a ∼50% loss of neuronal viability compared to control neurons (Figure 5A and 5B), as previously reported [21]–[23],[37].

Full-length human LRRK2 was packaged into a Herpes Simplex Virus (HSV) amplicon that co-expresses eGFP to generate an HSV-WT-LRRK2/CMV-eGFP amplicon. Expression of LRRK2 by the HSV amplicon causes similar neuronal toxicity to that of full-length WT LRRK2 transiently co-transfected into neurons with eGFP (Figure 5A and 5B), indicating that transient transfection is a reliable and valid method by which to assess LRRK2-induced toxicity. Thus, truncated LRRK2 proteins containing the GTPase domain produce similar neuronal toxicity to that induced by full-length WT LRRK2 implying that the GTPase domain may underlie the toxic effects of LRRK2.

To determine and compare the effects of truncated LRRK2 GTPase variants on neuronal viability, similar experiments were conducted with the GTP-COR-Kin LRRK2 fragment containing each mutation that was previously examined in the yeast model. Expression of the GTPase-active WT, R1398L and RQ/TG variants of LRRK2 induces a significant yet equivalent level of neuronal toxicity relative to control neurons characterized by a 10–15% loss of viability (Figure 5C and 5D). Expression of the LRRK2 GTPase-inactive variants, K1347A and T1348N, enhances neuronal toxicity compared to other GTPase variants with a ∼18% loss of viability for the K1347A variant and ∼23% loss for the T1348N variant that is significantly increased relative to the WT protein (Figure 5C and 5D). Thus, GTPase variants in truncated LRRK2 induce toxicity in neurons that closely parallel their toxic effects in yeast. Collectively, these data demonstrate the validity of the yeast model for accurately predicting the detrimental effects of truncated LRRK2 variants on neuronal viability. Taken together, these data reveal that alterations in GTPase activity contribute to LRRK2-induced neuronal toxicity.

### Expression of LRRK2 Causes Trafficking Defects in Neurons

**Figure 6:**
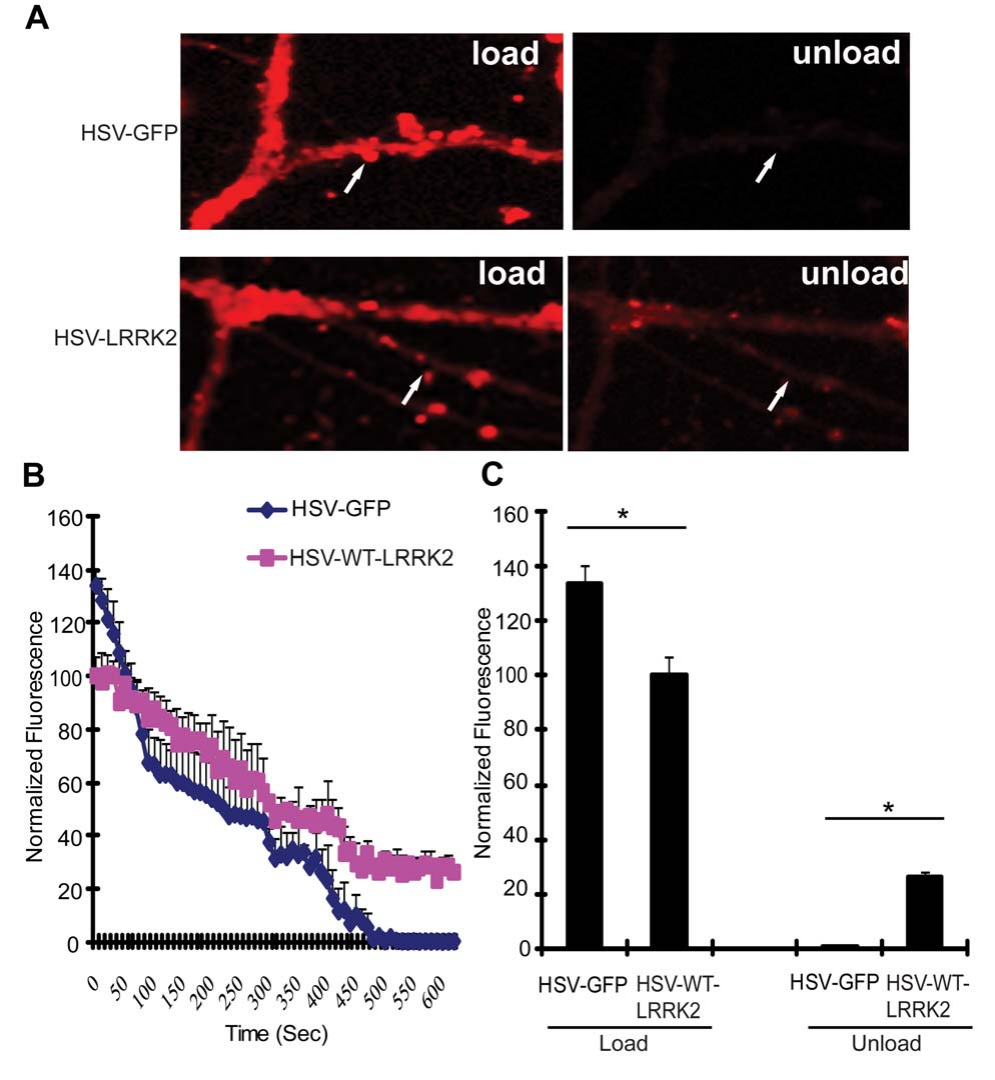
Expression of LRRK2 causes vesicular trafficking defects in neurons. (A) Hippocampal neuronal synapses transduced with HSV-WT-LRRK2/CMV-eGFP or HSV-PrPUC/CMV-eGFP as a control following FM4–64 dye loading by synaptic vesicle endocytosis and unloading by synaptic vesicle exocytosis. Arrows indicate synaptic boutons. (B) Dynamic real-time quantification of FM4–64 fluorescence intensity in synaptic boutons following dye loading and unloading over a 10 min period. Notice reduced initial loading and delayed release of the FM dye due to WT LRRK2 expression (C) Quantification of FM4–64 fluorescence intensity in synaptic boutons following dye loading at time point 0 sec and unloading at time point 480 sec. Data represent the mean ± SEM from three independent experiments (15 boutons for each experiment). Data were analyzed for statistical significance by two-tailed unpaired Student's *t*-test between HSV-FL-LRRK2 neurons and HSV-GFP control neurons (*P<0.01).

Since the LRRK2 yeast model indicates that truncated LRRK2 may function in vesicular trafficking pathways, including endocytosis, the effect of full-length human LRRK2 on endocytosis and exocytosis was monitored in primary neurons. Mouse hippocampal neurons at days *in vitro* (DIV) 12 were transduced with HSV-WT-LRRK2/CMV-eGFP or control virus and 48 hours later synaptic vesicle (SV) endocytosis and exocytosis were monitored by using the lipophilic fluorescent dye FM4–64. Neurons were first exposed to FM4–64 in the presence of 90 mM KCl, which depolarizes the nerve terminal and induces vesicular recycling and subsequent loading of FM4–64 by SV endocytosis. SV exocytosis was then monitored in real time by depolarizing the nerve terminals to unload the FM4–64 dye. Based on comparison of the mean fluorescence intensity values, the synaptic boutons of neurons carrying HSV-WT-LRRK2/CMV-eGFP display an approximate 1.34-fold decrease in loading of FM4–64 by endocytosis compared to the HSV-PrPUC/CMV-eGFP control (Figure 6A left panels, Figure 6B at time point ‘0’ sec, and Figure 6C: control, 133.99±5.897; WT LRRK2, 100.23±7.098). Following depolarization of the FM4–64-loaded SVs, the control boutons displayed about 99% unloading of FM4–64 after 8 mins, whereas the synaptic boutons overexpressing LRRK2 show delayed unloading with an approximate 72% decrease in FM4–64 signal (Figure 6A right panels, Figure 6B at time point ‘480’ secs, and Figure 6C: control, 0.886±0.851; LRRK2, 28.3±0.804). These data indicate that overexpression of full-length LRRK2 causes defects in both synaptic vesicle endocytosis and exocytosis in neurons consistent with the observation that overexpression of truncated LRRK2 variants in yeast perturbs vesicular trafficking pathways.

### A Genome-Wide Genetic Screen Identifies Modifiers of LRRK2 Toxicity in Yeast

**Figure 7:**
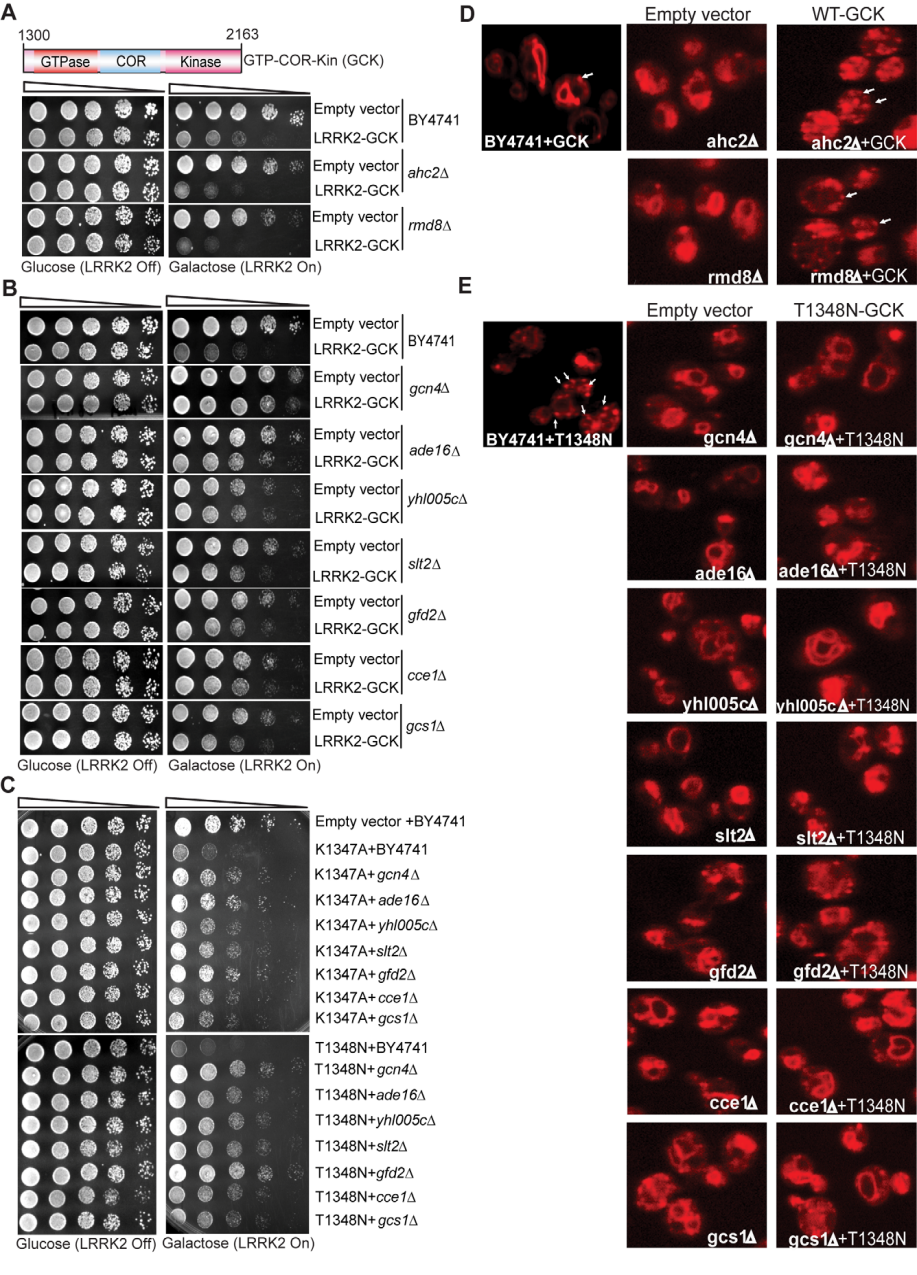
A genome-wide genetic screen identifies modifiers of LRRK2 toxicity in yeast. (A) Yeast gene deletion strains *ahc2Δ* and *rmd8Δ* markedly enhance LRRK2-induced toxicity in yeast compared to the WT strain. Yeast WT strain BY4741 and gene deletion strains *ahc2Δ* and *rmd8Δ* cells were transformed with galactose-inducible expression constructs containing the central GTP-COR-Kin fragment of WT LRRK2 or empty vector as a control. (B) Yeast gene deletion strains *gcn4Δ, ade16Δ, yhl005cΔ, slt2Δ, gfd2Δ, cce1Δ*, and *gcs1Δ* suppress LRRK2-induced toxicity in yeast compared to the WT strain. Yeast WT strain BY4741 and gene deletion strains *gcn4Δ, ade16Δ, yhl005cΔ, slt2Δ, gfd2Δ, cce1Δ*, and *gcs1Δ* cells were transformed with galactose-inducible expression constructs containing the central GTP-COR-Kin fragment of WT LRRK2 or empty vector as a control. (C) Yeast gene deletion strains *gcn4Δ, ade16Δ, yhl005cΔ, slt2Δ, gfd2Δ, cce1Δ*, and *gcs1Δ* markedly suppress LRRK2 mutant K1347A and T1348N-induced toxicity in yeast compared to the WT strain. Yeast WT strain BY4741 and gene deletion strains *gcn4Δ, ade16Δ, yhl005cΔ, slt2Δ, gfd2Δ, cce1Δ*, and *gcs1Δ* cells were transformed with galactose-inducible expression constructs containing K1347A and T1348N mutations in the central GTP-COR-Kin fragment of LRRK2 or empty vector as a control. Spotting experiments were conducted to examine the viability of yeast cells due to the expression of the LRRK2 fragment (A–C). Shown are five-fold serial dilutions (from left to right, as indicated by graded open box) starting with equal numbers of cells grown on media containing glucose (LRRK2 Off, left panel) or galactose (LRRK2 On, right panel). (D) Endocytosis of FM4–64 (red) was employed to monitor vesicular trafficking in yeast gene deletion strains *ahc2Δ* and *rmd8Δ* carrying the WT LRRK2 GTP-COR-Kin fragment. WT yeast cells expressing the GTP-COR-Kin fragment display normal ring-like vacuolar membrane staining (asterix) in addition to some punctate structures (arrow). Yeast gene deletion strains *ahc2Δ* and *rmd8Δ* expressing the GTP-COR-Kin fragment markedly disrupts FM4–64 vacuole localization with the appearance of multiple large punctate structures (arrows) whereas the *ahc2Δ* and *rmd8Δ* strains alone show normal vacuole staining. (E) Endocytosis of FM4–64 (red) was employed to monitor vesicular trafficking in yeast gene deletion strains *gcn4Δ, ade16Δ, yhl005cΔ, slt2Δ, gfd2Δ, cce1Δ*, and *gcs1Δ* carrying the LRRK2 GTP-COR-Kin mutant T1348N. WT yeast cells expressing the T1348N mutant display multiple large punctate structures (arrows). Yeast gene deletion strains *gcn4Δ, ade16Δ, yhl005cΔ, slt2Δ, gfd2Δ, cce1Δ*, and *gcs1Δ* carrying the mutant T1348N generally exhibit normal ring-like vacuolar membrane staining and a decrease in punctate structures.

To define mechanisms underlying LRRK2-induced cytotoxicity in yeast, we performed an unbiased genome-wide genetic screen to identify yeast genes that could suppress or enhance toxicity. A similar approach has been effective at identifying modifiers of α-synuclein or mutant huntingtin toxicity in yeast [39]. We mated a haploid query strain, harboring the galactose-inducible WT LRRK2 GTP-COR-Kin fragment, to a collection of ∼4,850 viable yeast deletion mutants. Following sporulation and haploid mutant selection, we isolated deletion mutants that suppressed or enhanced LRRK2 toxicity. Of 4,850 mutants screened, we identified 2 gene deletions that enhanced LRRK2 toxicity (Figure 7A, Table 1) and 7 deletions that suppressed toxicity (Figure 7B, Table 1). Furthermore, these 7 LRRK2 toxicity suppressors also suppressed toxicity induced by the LRRK2 mutants, K1347A and T1348N, in the GTP-COR-Kin fragment (Figure 7C). This set of yeast genes that modify LRRK2 cytotoxicty function in a number of diverse pathways including transcriptional regulation (*AHC1* (GenBank #CAA99213) and *GCN4* (GenBank #AAA34640)), MAP kinase signaling (*SLT2* (GenBank #AAB68912), small GTPase signaling (*GCS1* GenBank #CAA98805) and mitochondrial function (*CCE1* GenBank #AAB24906) (Table 1).

**Table 1:** Yeast deletion strains synthetically sick with truncated LRRK2 or suppressors of the toxicity induced by truncated LRRK2.

Strain	Homolog	Synthetically sick or suppressor	Function
*ahc2Δ*	No	Synthetically sick	Putative transcriptional regulator; proposed to be an Ada Histone acetyltransferase complex component
*rmd8Δ*	No	Synthetically sick	Cytosolic protein required for sporulation; Required for Meiotic nuclear Division
*gcn4Δ*	No	Suppressor	Basic leucine zipper (bZIP) transcriptional activator of amino acid biosynthetic genes in response to amino acid starvation
*ade16*Δ	No	Suppressor	Enzyme of ‘de novo’ purine biosynthesis containing both 5-aminoimidazole-4-carboxamide ribonucleotide transformylase and inosine monophosphate cyclohydrolase activities
*yhl005c*Δ	No	Suppressor	Unknown ORF, Dubious ORF
*slt2*Δ	Yes	Suppressor	Serine/threonine MAP kinase involved in regulating the maintenance of cell wall integrity and progression through the cell cycle; regulated by the PKC1-mediated signaling pathway
*gfd2Δ*	No	Suppressor	Identified as a high-copy suppressor of a dbp5 (Dead Box protein) mutation
*cce1Δ*	No	Suppressor	Mitochondrial cruciform cutting endonuclease, cleaves Holliday junctions formed during recombination of mitochondrial DNA
*gcs1Δ*	Yes	Suppressor	ADP-ribosylation factor GTPase activating protein 1 (ARFGAP1), involved in ER-Golgi transport; shares functional similarity with Glo3p

The homolog category indicates yeast genes with clear human homologs.

To further determine if these genetic modifiers enhance or suppress LRRK2 toxicity by modifying trafficking defects in yeast, we performed the FM4–64 assay in the two enhancer deletion mutants carrying WT GTP-COR-Kin fragment and the 7 suppressor deletion mutants carrying the most toxic LRRK2 mutant, T1348N. Interestingly, both enhancer mutants promote the endocytic trafficking defect induced by WT LRRK2 with an increase in the appearance of labeled punctate structures (Figure 7D) while the 7 suppressor mutants at least partially rescue the T1348N LRRK2-induced endocytic trafficking defect with the appearance of normal vacuolar membrane staining and a decrease in punctate structures (Figure 7E). These data suggest that the genetic modifiers can at least partially modulate vesicular trafficking pathways and genetically interact with LRRK2 to modify LRRK2-induced toxicity. Accordingly, these data suggest that vesicular trafficking defects in yeast underlie, in part, LRRK2-induced toxicity.

## Discussion

Here, we employ yeast cells to provide insight into the pathobiology of human LRRK2, a protein that is associated with autosomal dominant PD. A number of important conclusions can be derived from this yeast model. First, expression of LRRK2 fragments containing the GTPase domain markedly reduces the viability of yeast cells relative to other protein domains of LRRK2. The expression of full-length LRRK2 in yeast is problematic since it is highly insoluble and is sequestered into large cytoplasmic inclusions, which prevents its potential for inducing toxicity. Thus, it is only possible to develop a yeast model of LRRK2 pathobiology based upon protein domain fragments rather than the full-length protein. Second, consistent with a prominent role for the GTPase domain in mediating the toxic effects of LRRK2 in yeast, the viability of yeast cells can be modulated by alterations in GTPase activity due to several functional mutations. Notably, interfering with GTPase activity (i.e. GTP hydrolysis) but not GTP binding or kinase activity is sufficient to modify LRRK2-induced toxicity in yeast. The pathogenic mutants R1441C/G and Y1699C in full-length LRRK2 have significantly decreased GTPase activity consistent with the notion that reduced GTPase activity is toxic to cells. Importantly, however, pathogenic mutations associated with familial PD (i.e. R1441C and G2019S) do not influence the toxicity induced by truncated human LRRK2 in yeast which perhaps suggests that these mutations may only exert their deleterious effects in the context of full-length LRRK2 or in mammalian cells. Third, the expression of functional LRRK2 GTPase variants induce defects in the endocytic vesicular trafficking and autophagy pathways. Vesicular trafficking and autophagic defects closely correlate with the level of toxicity induced by each truncated GTPase variant suggesting that defects in trafficking may underlie LRRK2-induced toxicity in this model. Accordingly, genetic modifiers that suppress LRRK2 toxicity in yeast also suppress trafficking defects. Fourth, known suppressors of α-synuclein-induced cytotoxicity in yeast do not suppress LRRK2 toxicity suggesting that both proteins mediate their toxic effects through distinct trafficking pathways yet with the common outcome of impairing vesicular transport to the vacuole, the yeast equivalent of the mammalian lysosome. Thus, defects in vacuolar or lysosomal transport may commonly underlie the pathogenic effects of α-synuclein and LRRK2. Fifth, the toxic effects of truncated LRRK2 GTPase variants are similar between yeast and neuronal models of LRRK2 pathobiology and truncated or full-length LRRK2 cause similar endocytic trafficking defects in both yeast cells and neurons, respectively, suggesting that the yeast LRRK2 model is predictive of mammalian cells. Finally, a genome-wide genetic screen identified potent modifiers of LRRK2 toxicity in yeast, which may provide novel clues to the underlying mechanism of LRRK2-induced toxicity.

**Figure 8:**
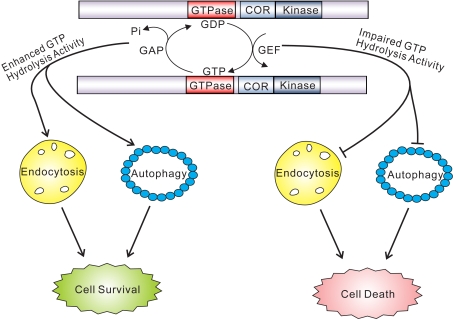
GTPase activity plays a key role in the pathobiology of LRRK2. GTP hydrolysis activity but not GTP binding or kinase activity is sufficient to modify toxicity induced by truncated human LRRK2 variants. Truncated GTPase variants with impaired GTP hydrolysis induce marked defects in the endocytic vesicular trafficking and autophagy pathways, which may underlie LRRK2-induced toxicity. Truncated GTPase variants with enhanced GTPase activity show reduced LRRK2-induced toxicity. Lines with arrows indicate promoting or activating effects while lines with blunt ends indicate inhibitory effects.

Neuronal toxicity induced by WT and pathogenic variants of full-length human LRRK2 critically requires intact GTP binding and kinase activity [21]–[23]. However, it has not yet been possible to distinguish, which, if any, of these activities actually mediates the downstream toxic effects of LRRK2 or whether they serve to auto-regulate an alternative function or effector domain of this protein. In yeast cells, the detrimental effects of expressing truncated LRRK2 variants are independent of kinase activity and are not influenced by two common pathogenic variants located either in the GTPase domain (i.e. R1441C) or the kinase domain (i.e. G2019S). Instead, toxicity is dependent on GTP hydrolysis activity, but not GTP binding activity. In the context of the central GTP-COR-Kin fragment of LRRK2 that is used here to explore the effects of GTPase variants, mutations that impair GDP/GTP binding and are thus GTPase-inactive promote toxicity, whereas mutations that produce a hyperactive GTPase partially reduce the toxic effects of LRRK2 (Figure 8). The lack of effect of kinase activity or pathogenic mutations on yeast toxicity induced by truncated human LRRK2, might suggest that they require the full-length protein or a mammalian cellular context to exert their effects on LRRK2-induced toxicity.

In the context of full-length LRRK2, the K1347A and T1348N mutations prevent GTP binding and are GTPase-inactive but also impair kinase activity, which partially prevents LRRK2-induced neuronal toxicity [14], [15], [19]–[23]. The RQ/TG mutation produces a Ras-like GTPase that also has impaired kinase activity owing to its increased turnover of GTP [15],[40], a feature reflected in our yeast model. The R1398L mutation also promotes GTP hydrolysis and accordingly we observe that introduction of this mutation into full-length LRRK2 produces a kinase-inactive variant (data not shown). The effects of the hyperactive GTPase mutants, RQ/TG and R1398L, on neuronal toxicity induced by full-length LRRK2 have not been defined, but they are likely to be protective due to their impairment of kinase activity and enhancement of GTPase activity. Both R1398L and RQ/TG mutants are capable of hydrolyzing GTP but their affinity for binding to GTP is reduced suggesting that they most likely predominate in a GDP-bound inactive state. It is likely that GTPase-inactive variants of LRRK2 induce greater toxicity in yeast through a novel gain-of-function mechanism by interfering with a pathway or process, or sequestering one or more proteins, critical for yeast survival or growth. A dominant-negative mechanism for LRRK2-induced toxicity is unlikely since yeast do not contain an obvious ortholog of human LRRK2. While the truncated LRRK2 protein used herein does not behave in a manner identical to full-length protein with regards to the regulation of cytotoxicity in yeast or neurons, it instead reveals a fundamental contribution of the GTPase domain and particularly GTP hydrolysis activity in mediating the toxic effects of LRRK2. A major challenge in future experiments will therefore involve dissecting the precise contribution of GTPase activity, vesicular trafficking pathways and genetic modifiers to neuronal toxicity induced by full-length LRRK2 variants.

The fact that LRRK2 kinase activity plays no role in yeast toxicity allowed us to reveal instead a major role for the GTPase domain in toxicity induced by truncated LRRK2 in both yeast and neurons. Fragments of other disease-causing gene products, such as in Huntington's disease or other poly-glutamine repeat disorders [41]–[44], TDP-43opathies [45],[46] and α-synucleinopathies [47],[48] play prominent roles in neurodegeneration due to the pathogenic generation of these truncated proteins. Interestingly, putative truncation fragments containing the LRRK2 GTPase domain have been identified in PD brains [3],[49]. In addition, E1874stop is a LRRK2 pathogenic mutation in which the protein lacks the kinase and WD40 domains [50]. Thus, understanding whether GTPase domain-containing truncated LRRK2 proteins are important for disease pathogenesis and how the GTPase domain modulates full-length LRRK2 activity are important avenues of investigation. Moreover, since the truncated GTPase domain-containing LRRK2 constructs are toxic in the absence of kinase activity, caution may be warranted by solely focusing on kinase inhibition as a therapeutic target for preventing LRRK2-induced neurodegeneration. Indeed, the GTPase-inactive K1347A mutant in the context of the full-length G2019S LRRK2 protein only partially rescues LRRK2 toxicity despite completely inhibiting kinase activity suggesting that perturbations in the GTPase domain may have deleterious consequences in the setting of full-length LRRK2 independent of kinase activity [21].

The mechanism by which truncated human LRRK2 is toxic to yeast is unclear. The GTPase domain would appear to play a key role in mediating toxicity but other protein domains may also contribute. LRRK2-induced defects in endocytic vesicular trafficking and autophagy may underlie toxicity in yeast, an observation supported by the actions of genetic modifiers of toxicity on vesicular trafficking. Consistent with the yeast LRRK2 model, full-length LRRK2 causes defects in synaptic vesicle endocytosis and exocytosis in neurons. Many other observations suggest that full-length LRRK2 may play a role in vesicular trafficking in mammalian neurons. LRRK2 is localized exclusively to a wide range of vesicular and membranous structures in neurons, including lysosomes, endosomes, multivesicular bodies, the ER, Golgi, mitochondria and microtubule transport vesicles [51],[52]. The G2019S variant promotes the formation of LRRK2-positive axonal inclusions in neurons that are membrane-bound and contain swollen lysosomes, distended mitochondria associated with vacuoles, multivesicular bodies and disrupted cytoskeletal components [24], perhaps suggestive of disruption of normal vesicular trafficking.

Consistent with our studies, a potential role for LRRK2 in endocytosis has recently been described [53]. LRRK2 interacts and co-localizes with Rab5B on synaptic vesicles. Knockdown or over-expression of LRRK2 in rodent primary neurons impairs synaptic vesicle endocytosis that can be rescued by over-expression of Rab5B [53], a GTPase involved in the early endocytic pathway from plasma membrane to early endosome. Studies in *C.elegans* with the human LRRK2 homolog, LRK-1, reveal a role for this protein in regulating the proper transport of synaptic vesicles to axonal regions possibly by acting at the *trans*-Golgi network to sort vesicles away from an alternative dendrite-specific transport mechanism [54]. Thus, in yeast it is likely that truncated LRRK2 interferes with the endocytic trafficking and autophagic pathways through functionally interacting or competing with key proteins involved in as yet unspecified steps during the transport of vesicles or their protein cargo from the plasma membrane and/or autophagosomes to the vacuole.

LRRK2-associated neurite shortening induced by the G2019S variant may be mediated at least in part by autophagy, since it is associated with the development of autophagic vacuoles and can be reversed by impairing autophagy and potentiated by activating autophagy [55]. In yeast, macroautophagy constitutes an additional pathway for vacuolar transport involving the formation and delivery of large double-membrane vesicles termed autophagosomes containing cytoplasmic constituents and organelles to the vacuole for degradation and recycling. The macroautophagy pathway is also perturbed in our yeast LRRK2 model in addition to the endocytic vesicular trafficking pathway. Consistent with our studies, a potential role for LRRK2 in the endosomal-autophagic pathway has recently been described [56]. Collectively, the observations from neuronal and yeast models tend to support a role for LRRK2 in regulating the sorting or transport of vesicles via endocytosis or autophagic pathways that possibly converge on the vacuole/lysosome (Figure 8). Further study of the biology and pathobiology of LRRK2 in regulating vacuolar/lysosomal function and dynamics may prove particularly insightful. In particular, it will be important to clarify whether derangements in endocytic and autophagic trafficking pathways critically underlie the neuronal toxicity induced by disease-associated full-length LRRK2 variants and the mechanism(s) involved in this pathologic process.

The observation that GTPase activity plays a key role in LRRK2 toxicity may prove highly useful in dissecting the molecular mechanism(s) underlying LRRK2-induced cytotoxicity and in the identification of genes or small molecules that can directly or indirectly modulate the GTPase activity of LRRK2. The relevance of such an approach would be to identify modifiers of GTPase activity that would additionally prevent kinase activation as an alternative novel strategy to inhibit the pathogenic effects of LRRK2. The key demonstration that truncated LRRK2 variants have similar effects on the viability of both yeast and neuronal cells suggests that this yeast LRRK2 model could be predictive for identifying genetic and chemical modifiers of conserved pathways, processes or proteins that are relevant for LRRK2-induced toxicity in neuronal models including human neuronal models derived from iPS cells.

Our genome-wide genetic screen to identify suppressors and/or synthetic sick or lethal interactions of LRRK2-induced toxicity in yeast identified modifiers in a number of diverse pathways including genes that are involved in transcriptional regulation, MAP kinase signaling, small GTPase signaling and mitochondrial function. These genes may play important roles in the pathobiology of LRRK2-linked PD. Notably, two of the deletion suppressors have human homologs. *SLT2* has four human homologs, which are serine/threonine MAP kinases MAPK1, 3, 11 and 14 involved in the initiation of translation, meiosis, mitosis, and postmitotic functions in differentiated cells. In addition they mediate their response via activation by environmental stress, pro-inflammatory cytokines and lipopolysaccharide by phosphorylating a number of substrates. The human homolog of *GCS1* is ADP-ribosylation factor GTPase activating protein 1 (ARFGAP1) which plays a role in membrane trafficking and/or vesicle transport. These deletion suppressors may prove to be attractive drug targets and they may provide important insight into the function of LRRK2.

In summary, our results provide evidence that the GTPase domain may contribute to LRRK2-induced toxicity, with enhanced GTP hydrolysis leading to reduced LRRK2 toxicity and impaired GTP hydrolysis leading to enhanced LRRK2 toxicity. In addition, our identification of genetic modifiers of LRRK2-induced toxicity in yeast provides important clues to proteins or pathways that may play key roles in mediating LRRK2-induced toxicity in higher organisms.

## Materials and Methods

### Ethics Statement

All procedures involving animals were approved by and conformed to the guidelines of the Institutional Animal Care Committee of Johns Hopkins University.

### Yeast Strains and Genetic Procedures

Yeast haploid strain BY4741 (*MATa, his3Δ1, leu2Δ0, met15Δ0, ura3Δ0*) obtained from Open Biosystems (Huntsville, AL) was used throughout this study. For the yeast genetic screen, the LRRK2 query strain was constructed in Y7092 (*MAT*α, *can1Δ::STE2pr-Sp_his5, lyp1Δ, his3Δ1, leu2Δ0, ura3Δ0, met15Δ0*). Yeast manipulations were performed and media were prepared using standard procedures. Transformations of yeast were performed using a standard high efficiency lithium acetate procedure [57]. Yeast cells carrying galactose-inducible expression constructs were routinely grown in YPD or synthetic complete media lacking uracil (SC-URA) containing glucose (2% dextrose) to repress the *GAL1* promoter. Yeast cells were pre-grown in SC-URA containing 2% raffinose (no repression of the *GAL1* promoter) prior to growth in medium containing 2% galactose (to induce the *GAL1* promoter), to allow rapid, synchronous induction of expression. Yeast cells that were co-transformed with two galactose-inducible constructs (with *URA3* or *LEU2* markers) were grown in SC-URA/-LEU to select for both plasmids.

### Plasmid Generation and Antibodies

Human LRRK2 fragment cDNAs were amplified from a pcDNA3.1-LRRK2-Myc-His vector [18] by PCR with primer pairs specific for the different domains of LRRK2 (refer to Figure 1A) with incorporation of an optimal yeast Kozak sequence (AAAAATGTCT) surrounding an ATG start codon (underlined). PCR products were first cloned into the pCR2.1-TOPO TA cloning vector (Invitrogen, Carlsbad, CA) before subcloning into the *GAL1* promoter-based yeast expression vector pYES2/CT (2 µ ori, *URA3*; Invitrogen) or p416GAL (CEN ori, *URA3*; kindly provided by Martin Funk [58]) containing a C-terminal V5 tag, or into mammalian expression vector pcDNA3.1-Myc-His (Invitrogen) via *BamH*I and *Xho*I restriction sites. Missense mutations were introduced into the GTP-COR-Kin fragment of LRRK2 by PCR-mediated, site-directed mutagenesis, using the QuickChange XL kit (Stratagene), followed by sequencing of the entire cDNA to confirm their correct incorporation. Candidate genes (*YPT1*, *YKT6* and *HSP31*) were amplified from yeast genomic DNA by PCR to also introduce a C-terminal V5 tag and stop codon, and resulting cDNAs were cloned into the *GAL1* promoter-based yeast expression vector p425GAL (2 µ ori, *LEU2*, kindly provided by Martin Funk [58]. All cDNAs were subjected to DNA sequencing to confirm their integrity.

Mouse monoclonal antibodies to yeast 3-phosphoglycerate kinase (PGK, clone 22C5), anti-V5 and anti-V5-HRP were obtained from Invitrogen. Mouse monoclonal anti-myc antibody (clone 9E10) was purchased from Roche Biochemicals. Rabbit polyclonal anti-GFP antibody (NB 600-303) was obtained from Novus Biologicals. HRP-linked anti-rabbit or anti-mouse IgG antibodies were obtained from Jackson ImmunoResearch Labs (West Grove, PA). AlexaFluor-488 anti-mouse IgG and AlexaFluor-594 anti-rabbit IgG antibodies were from Molecular Probes. Human LRRK2-specific antibody JH5517 has been described previously [18],[59].

### Yeast Cell Viability Assays

#### Spotting experiments

Cells carrying galactose-inducible expression constructs were grown overnight at 30°C in liquid media (SC-URA or SC-URA/-LEU) containing raffinose to log phase, followed by growth in media containing galactose for a further 6 hrs. Cultures were then normalized for OD_600 nm_, serially diluted (5-fold) and spotted onto plates containing solid media (SC-URA or SC-URA/-LEU) with either glucose or galactose as the sole carbon source. Cells were grown at 30°C for at least 2 days before imaging.

*Growth curves*: Cells were grown overnight in synthetic medium (SC-URA or SC-URA/-LEU) containing raffinose and then diluted to an OD_600 nm_ of 0.1 before inducing expression in media by addition of galactose. OD_600 nm_ measurements were taken at indicated time points over 18 hrs.

### Reverse-Transcription (RT)–PCR

Yeast cells carrying galactose-inducible LRRK2 constructs were grown and induced as described for spotting experiments. Total RNA was isolated from yeast cells by hot phenol extraction [60] and further purified using the Qiagen RNEasy Mini kit (Qiagen). Total RNA concentrations were determined with a Nanodrop spectrophotometer (Nanodrop Technologies) prior to RT-PCR. cDNAs were generated from total RNA using the OneStep RT-PCR kit (Qiagen) and oligo-d(T). PCR was conducted on equal quantities of mRNA-derived cDNAs for 25 cycles with *LRRK2*-specific primers located within the kinase domain (Forward: 5′-CCAGATCAACCAAGGCTCAC-3′, Reverse: 5′-CCTGCTGTTGTGATGTGTAG-3′) or yeast actin (*ACT1*) primers (Forward: 5′-TCGATTTGGCCGGTAGAGATT-3′, Reverse: 5′-AAGATGGAGCCAAAGCGGTGATT-3′) as a loading control.

### GTP Binding Assay

Yeast cells carrying galactose-inducible LRRK2 constructs were grown and induced as described for spotting experiments. Total proteins were extracted from yeast by a standard method using glass bead lysis. Briefly, yeast cells were pelleted and lysed in 1 ml lysis buffer (1 X PBS, pH 7.4, 1% NP-40, 1 x phosphatase inhibitor cocktail 1 and 2 [Sigma-Aldrich], 1 x Complete mini protease inhibitor cocktail [Roche]) by vigorous shaking with glass beads at 4°C for 15 min and lysates were clarified by centrifugation at 17,500×*g* for 10 min at 4°C. Supernatants were incubated with 50 µl γ-aminohexyl-GTP-sepharose bead suspension (Jena Bioscience, Jena, Germany) by rotating at 4°C for 2 hr. The sepharose beads were sequentially pelleted and washed twice in wash buffer (1 X PBS, pH 7.4, 1% Triton X-100) and twice with PBS alone. GTP-bound proteins were eluted into 50 µl Laemmli sample buffer (BioRad) containing 5% 2-mercaptoethanol by heating for 10 min at 95°C. GTP-bound proteins or input controls (0.1% total lysate) were resolved by SDS–PAGE and subjected to Western blot analysis with anti-V5 antibody. Bands were visualized by enhanced chemiluminescence (Amersham). Quantification of protein expression was performed using densitometry analysis software (AlphaImager, Alpha Innotech Corp.).

### GTPase Activity Assay

GTP hydrolysis activity was measured by monitoring the release of free γ-phosphate (P_i_) from GTP. Briefly, total proteins were prepared from yeast cells carrying galactose-inducible LRRK2 constructs as described for GTP binding assays. Soluble lysates were subjected to immunoprecipitation with anti-V5 antibody (1 µg) pre-incubated with 50 µl Protein G Dynabeads (Invitrogen) by rotating at 4°C overnight. Dynabeads were stringently washed 5x with lysis buffer before being subjected to GTPase activity assay in 96-well plates using the colorimetric GTPase assay kit (Innova Biosciences, Cambridge, UK) as per manufacturers instructions to measure the concentration of free P_i_ with absorbance measured at 590–660 nm. LRRK2 immunoprecipitates (anti-V5) were also analyzed by Western blot analysis with anti-V5 antibody to quantify the input levels of each LRRK2 variant for normalization purposes. Densitometric analysis was conducted on protein bands using appropriate software (AlphaImager, Alpha Innotech Corp.). A similar procedure was employed for myc-tagged full-length human LRRK2 variants derived from HEK-293T cells to measure LRRK2 GTPase activity.

### HSV-LRRK2

The HSV amplicon platform was utilized to generate HSV-LRRK2 expression vectors containing full-length human LRRK2 [61].

### Transmission Electron Microscopy (TEM)

TEM was performed on yeast cells expressing truncated LRRK2 variants as previously described [45],[62] at the Integrated Imaging Center, Johns Hopkins University.

### FM4–64 Assay in Yeast

Yeast cells carrying galactose-inducible LRRK2 constructs were grown and induced as described for spotting experiments. Following galactose induction for 6 hrs, 1 ml of culture was harvested by brief centrifugation, resuspended in SC-URA media containing galactose and 40 µM FM4–64 red fluorescent dye (Molecular Probes) and incubated at 30°C for 20 minutes to allow dye internalization by endocytosis. Cells were washed once in SC-URA media containing galactose before being dispersed and mounted onto microscope slides. Imaging of red fluorescence was conducted on a Zeiss LSM510 live confocal system.

### FM4–64 Assay in Neurons

Mouse primary hippocampal neurons (E15–16) were transduced by HSV-WT-LRRK2/CMV-eGFP and HSVPrPUC/CMV-eGFP virus at DIV 12. After 48 hour transduction, cells were mounted in a laminar-flow perfusion chamber on the stage of a custom-built laser scanning confocal microscope using a calcium containing buffer (Solution B: 119 mM NaCl, 2.5 mM KCL, 4 mM MgCl_2_, 30 mM Glucose, 25 mM HEPES, 2 mM CaCl_2_). After gently removing Solution B cells were then continuously perfused with Solution A (Solution B without CaCl_2_). The first stimulus was then applied with FM dye containing Solution D (90 mM KCl, 29 mM NaCl, 2 mM CaCl_2_, 2 mM MgCl_2_, 30 mM Glucose, 25 mM HEPES and 15 µM FM4–64, Molecular Probes) for 2 min. This step leads to presynaptic release, vesicle fusion and dye incorporation by synaptic vesicle endocytosis. Cells were then washed by perfusion with Solution A for up to 10 min to minimize background staining. After gently aspirating Solution A, Solution C (Solution D without FM dye) is applied to cause release of the FM dye by synaptic vesicle exocytosis. Images were acquired every 10 sec with a CCD camera. The fluorescence intensity of manually designated pre-synaptic regions was quantified.

### Primary Cortical Neuronal Cultures and Viability Assay

Primary cortical neuronal cultures were prepared and transiently transfected with LRRK2 or eGFP expression constructs as described previously [22],[37]. Briefly, cortices were dissected from embryonic day 15–16 fetal mice (CD1 strain), dissociated by a 12 min digestion in TrypLE (Invitrogen), and neurons were seeded into 24-well plates coated with poly-L-ornithine. Neurons were routinely maintained in Neurobasal media (Invitrogen) containing 2 mM L-glutamine and 2% B27 supplement at 37°C in a 7% CO_2_ humidified incubator. Glial cell growth was inhibited by addition of 5-fluoro-20-deoxyuridine (5F2DU, 30 µM, Sigma) to the media on days *in vitro* (DIV) 4. Media was replaced once every third day. At DIV 10, neurons represented >90% of total cells in the culture. To assess LRRK2-induced toxicity, neurons at DIV 10 were transiently co-transfected with LRRK2 and eGFP expression constructs at a molar ratio 10∶1, respectively, using Lipofectamine 2000 reagent (Invitrogen) according to the manufacturer recommendations. At 48 hrs post-transfection (DIV 12), live fluorescent images were collected on a Zeiss Automatic stage microscope with Axiovision 6.0 software. Neurons with obvious neurite process and/or nuclear fragmentation were counted as non-viable cells by investigators blinded to the identity of the experiment. For each independent experiment, the percent viability of eGFP-positive neurons (*n* = 200) was determined and data are presented as a percent of control neurons transfected with eGFP alone. Neurons were subsequently fixed with 4% paraformaldehyde and immunocytochemistry was conducted with anti-myc (Roche) and anti-GFP (Novus Biologicals) antibodies and appropriate fluorescent secondary antibodies. LRRK2 expression was confirmed in >95% of eGFP-positive neurons (Figure S5A).

### TUNEL staining

The above transfected neurons were fixed in 4% paraformaldehyde (PFA) after 48 hrs transfection. TUNEL staining was performed using the *In Situ* Cell Death Detection Kit (Roche) as per the manufacturer's instructions.

### Immunocytochemistry

Yeast cells carrying galactose-inducible LRRK2 constructs were grown and induced as described for spotting experiments. Following galactose induction for 6 hrs, 1 ml culture was harvested by brief centrifugation, and fixed in 4% formaldehyde/PBS for 1 hr. Cell walls were digested by incubation with Zymolyase 20T solution (ICN Biochemicals), as recommended. Following permeabilization, cells were gently washed twice in KS solution (100 mM potassium phosphate pH 7.0, 1 M sorbitol), and then resuspended in KS solution. Immunostaining with mouse monoclonal anti-V5 antibody (Invitrogen) and AlexaFluor-488 anti-mouse IgG (Molecular Probes) was conducted as previously described [63] Cells were dispersed onto microscope slides and mounted using Vectashield mounting medium containing DAPI (Vector Laboratories) for nuclear visualization. Fluorescent images were collected on a Zeiss Automatic stage microscope with Axiovision 6.0 software.

### Yeast Genome-Wide Genetic Screen

The yeast LRRK2 toxicity modifier screen was performed using synthetic genetic array (SGA) analysis [64] as previously described [65]. We used a Singer RoToR HAD yeast pinning robot for manipulating yeast colonies at high density. A MATα yeast haploid query strain, Y7092, carrying WT LRRK2 GTP-COR-Kin fragment was mated with a haploid yeast gene deletion collection of 4850 viable mutants, sporulated and then underwent selection for haploid mutants that also harbored the LRRK2 plasmid on solid media containing G418 and lacking uracil. Haploid deletion mutants that also carried the LRRK2 plasmid were identified on selectable media containing glucose and then the expression of LRRK2 was induced by growth on galactose media. After comparing colony sizes on galactose plates to those on glucose plates and normalizing for differences in the growth of deletion mutants between carbon sources, genes that suppressed or enhanced LRRK2 toxicity were identified. Initial hits from the screen were independently verified by fresh transformations and spotting assays.

## Supporting Information

Figure S1 Expression of full-length human LRRK2 in yeast cells. (A) Cell viability assay was employed to analyze the effect of high copy and low copy expression of full-length WT LRRK2 or high copy expression of G2019S LRRK2 on yeast growth compared to control cells (empty vector). Shown are five-fold serial dilutions (from left to right, as indicated by graded open box) starting with equal numbers of cells spotted onto glucose (LRRK2 Off, left panel) or galactose (LRRK2 On, right panel) media. (B) Growth curve analysis in liquid media containing galactose was used to measure the growth rate of yeast cells expressing full-length LRRK2 variants or containing empty vector. (C) Expression of full-length LRRK2 (WT or G2019S) in yeast cells was detected by Western blot analysis (SDS-PAGE with 8% Urea) with LRRK2-specific antibody (JH5517) on urea-soluble proteins extracted from yeast following galactose induction for 6 or 12 hrs or just prior to induction (0 hrs) in liquid media. Full-length human LRRK2 transiently expressed in HEK-293 cells was used as a positive control. Note that full-length LRRK2 expressed in yeast cells is highly insoluble and accumulates at the top of the stacking gel whereas LRRK2 expressed in HEK-293 cells migrates normally at ∼260 kDa. (D) mRNA expression levels of full-length LRRK2 in yeast cells were detected by RT-PCR with LRRK2-specific primers and actin (ACT1) primers as a loading control. (E) Immunofluorescent localization of full-length human LRRK2 (WT or G2019S) expressed in yeast cells. LRRK2 subcellular localization was revealed by immunostaining cells with a human LRRK2-specific antibody (JH5517) following galactose induction. Arrows indicate large LRRK2-positive cytoplasmic inclusions that are absent from control cells (empty vector).
(3.20 MB TIF)

Figure S2 Localization of truncated LRRK2 variants in yeast. LRRK2 domain fragments (A) and LRRK2 GTPase functional variants in the GTP-COR-Kin fragment (B) exhibit similar diffuse cytoplasmic localization patterns in yeast cells. Fluorescence microscopy was employed to visualize the subcellular localization of each LRRK2 construct following galactose induction. Cells were stained with anti-V5 antibody (green) and counterstained with DAPI (blue) to label nuclei. The overlay of LRRK2 and DAPI fluorescence is also indicated.
(14.70 MB TIF)

Figure S3 Kinase-modifying mutations fail to influence LRRK2-induced toxicity in yeast. Kinase-modifying mutations were introduced into the kinase domain of the GTP-COR-Kin LRRK2 fragment, including two pathogenic variants that enhance kinase activity (G2019S and R1441C) and two kinase-impaired mutations (K1906M and TripKIN [T2031A/S2032A/T2035A, representing three putative autophosphorylation sites in the kinase activation loop]). Cell viability assay was employed to examine the effects of modulating kinase activity on the viability of yeast cells. Shown are five-fold serial dilutions (from left to right, as indicated by graded open box) starting with equal numbers of cells spotted onto glucose (repressed, off, left panel) or galactose (induced, on, right panel) media.
(1.34 MB TIF)

Figure S4 Candidate genetic screen for suppressors of LRRK2-induced toxicity in yeast. (A) Cell viability assay for three candidate genes (HSP31, YPT1, and YKT6) transformed either alone or together with WT LRRK2 (GTP-COR-Kin fragment). Empty vector was used as a control for viability. Shown are five-fold serial dilutions (from left to right, as indicated by graded open box) starting with equal numbers of cells spotted onto glucose (repressed, LRRK2 Off, left panel) or galactose (induced, LRRK2 On, right panel) media. Below, Western blot analysis of total proteins from each yeast transformant following galactose induction probed with anti-V5 antibody to confirm the expression of LRRK2 and each candidate interactor protein. (B) Cell viability assay for three candidate genes co-transformed with LRRK2 GTPase variants, K1347A or T1348N, in the GTP-COR-Kin fragment, compared to single transformation of LRRK2 alone or empty vector as controls. Shown are five-fold serial dilutions (from left to right, as indicated by graded open box) starting with equal numbers of cells spotted onto glucose or galactose media.
(3.16 MB TIF)

Figure S5 Representative images demonstrating the co-expression of LRRK2 and eGFP, neuronal viability and confirmation of neuronal type in the LRRK2-induced neuronal toxicity assay. (A) LRRK2 is expressed in >95% eGFP-positive neurons when co-transfected with eGFP into primary neurons at a molar ratio of 10∶1. Neurons were co-stained by anti-MYC (LRRK2) and anti-GFP antibodies after 48 hr co-transfection with LRRK2 and eGFP plasmids. (B) Neuronal viability was confirmed by TUNEL staining. Neurons were stained by TUNEL and with anti-GFP antibody after 48 hr co-transfection of LRRK2 and eGFP. The arrow indicates a non-viable eGFP-positive neuron expressing G2019S LRRK2 that also exhibits a TUNEL-positive nucleus. eGFP-positive neurons carrying empty vector or viable neurons expressing G2019S LRRK2 are negative for nuclear TUNEL staining. (C) Confirmation of LRRK2 expression in MAP2-positive cortical neurons. Neurons were co-stained with anti-MAP2 and anti-GFP antibodies after 48 hr co-transfection of LRRK2 and eGFP plasmids at a 10∶1 molar ratio. eGFP-positive neurons expressing WT LRRK2 are positive for the neuronal marker MAP2.
(18.51 MB TIF)
