# Supplementary figures and images for: GTPase Activity Plays a Key Role in the Pathobiology of LRRK2
Source: PLoS Genet. 2010 Apr 8;6(4):e1000902. doi: 10.1371/journal.pgen.1000902 (PMC2851569; doi:10.1371/journal.pgen.1000902)

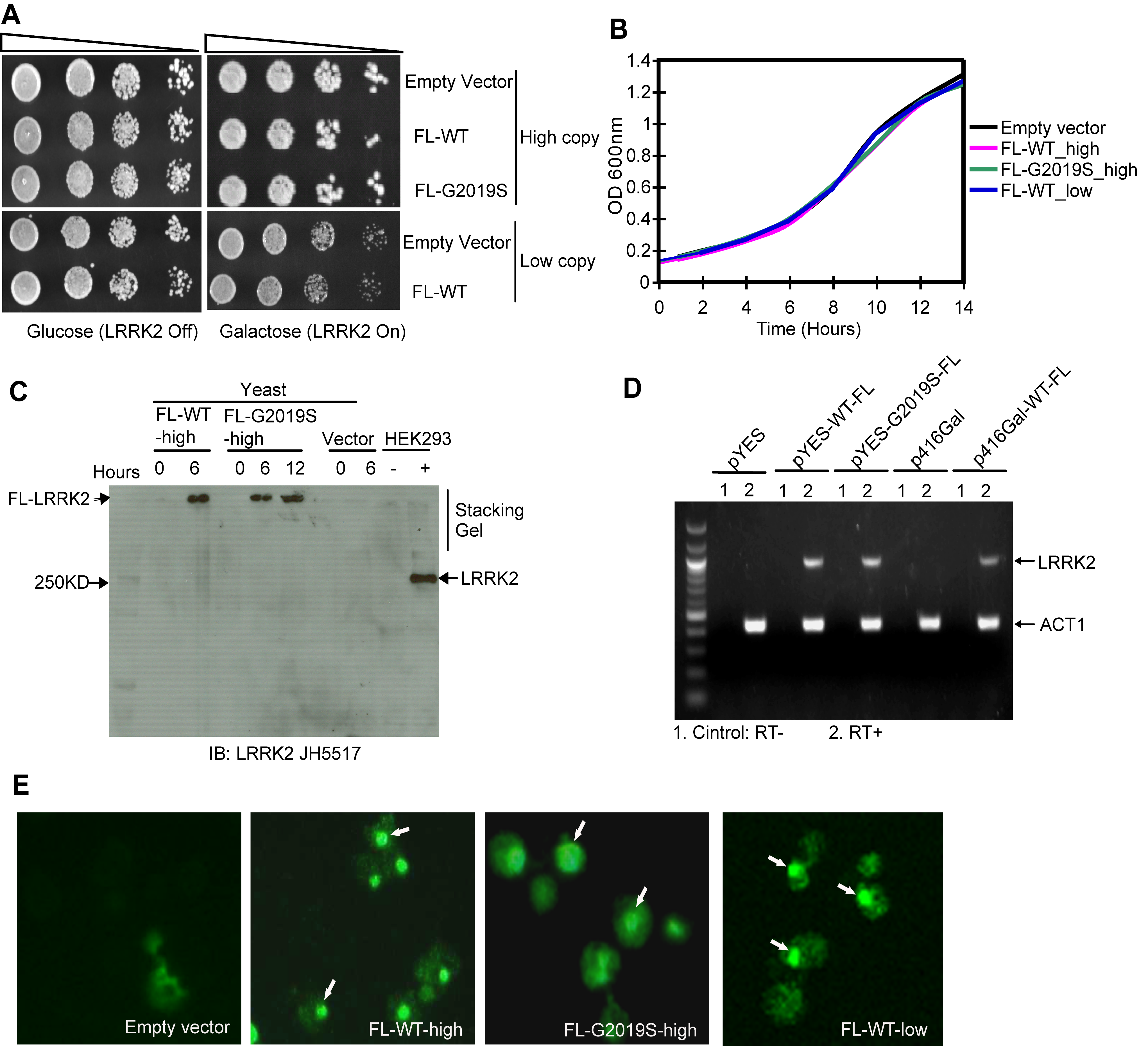

Supplement: Figure S1 — Expression of full-length human LRRK2 in yeast cells. (A) Cell viability assay was employed to analyze the effect of high copy and low copy expression of full-length WT LRRK2 or high copy expression of G2019S LRRK2 on yeast growth compared to control cells (empty vector). Shown are five-fold serial dilutions (from left to right, as indicated by graded open box) starting with equal numbers of cells spotted onto glucose (LRRK2 Off, left panel) or galactose (LRRK2 On, right panel) media. (B) Growth curve analysis in liquid media containing galactose was used to measure the growth rate of yeast cells expressing full-length LRRK2 variants or containing empty vector. (C) Expression of full-length LRRK2 (WT or G2019S) in yeast cells was detected by Western blot analysis (SDS-PAGE with 8% Urea) with LRRK2-specific antibody (JH5517) on urea-soluble proteins extracted from yeast following galactose induction for 6 or 12 hrs or just prior to induction (0 hrs) in liquid media. Full-length human LRRK2 transiently expressed in HEK-293 cells was used as a positive control. Note that full-length LRRK2 expressed in yeast cells is highly insoluble and accumulates at the top of the stacking gel whereas LRRK2 expressed in HEK-293 cells migrates normally at ∼260 kDa. (D) mRNA expression levels of full-length LRRK2 in yeast cells were detected by RT-PCR with LRRK2-specific primers and actin (ACT1) primers as a loading control. (E) Immunofluorescent localization of full-length human LRRK2 (WT or G2019S) expressed in yeast cells. LRRK2 subcellular localization was revealed by immunostaining cells with a human LRRK2-specific antibody (JH5517) following galactose induction. Arrows indicate large LRRK2-positive cytoplasmic inclusions that are absent from control cells (empty vector). (3.20 MB TIF) [file pgen.1000902.s001.tif]

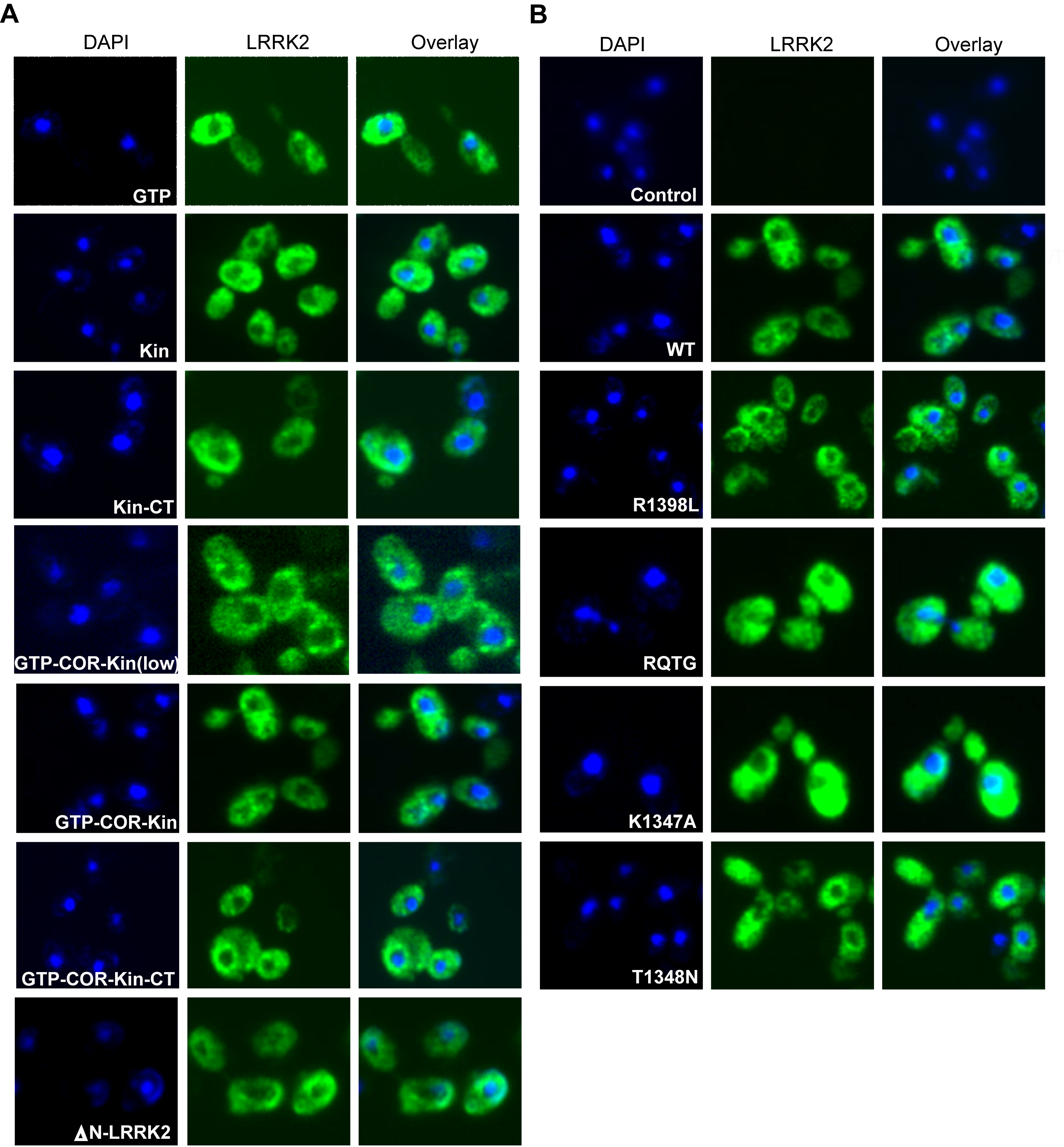

Supplement: Figure S2 — Localization of truncated LRRK2 variants in yeast. LRRK2 domain fragments (A) and LRRK2 GTPase functional variants in the GTP-COR-Kin fragment (B) exhibit similar diffuse cytoplasmic localization patterns in yeast cells. Fluorescence microscopy was employed to visualize the subcellular localization of each LRRK2 construct following galactose induction. Cells were stained with anti-V5 antibody (green) and counterstained with DAPI (blue) to label nuclei. The overlay of LRRK2 and DAPI fluorescence is also indicated. (14.70 MB TIF) [file pgen.1000902.s002.tif]

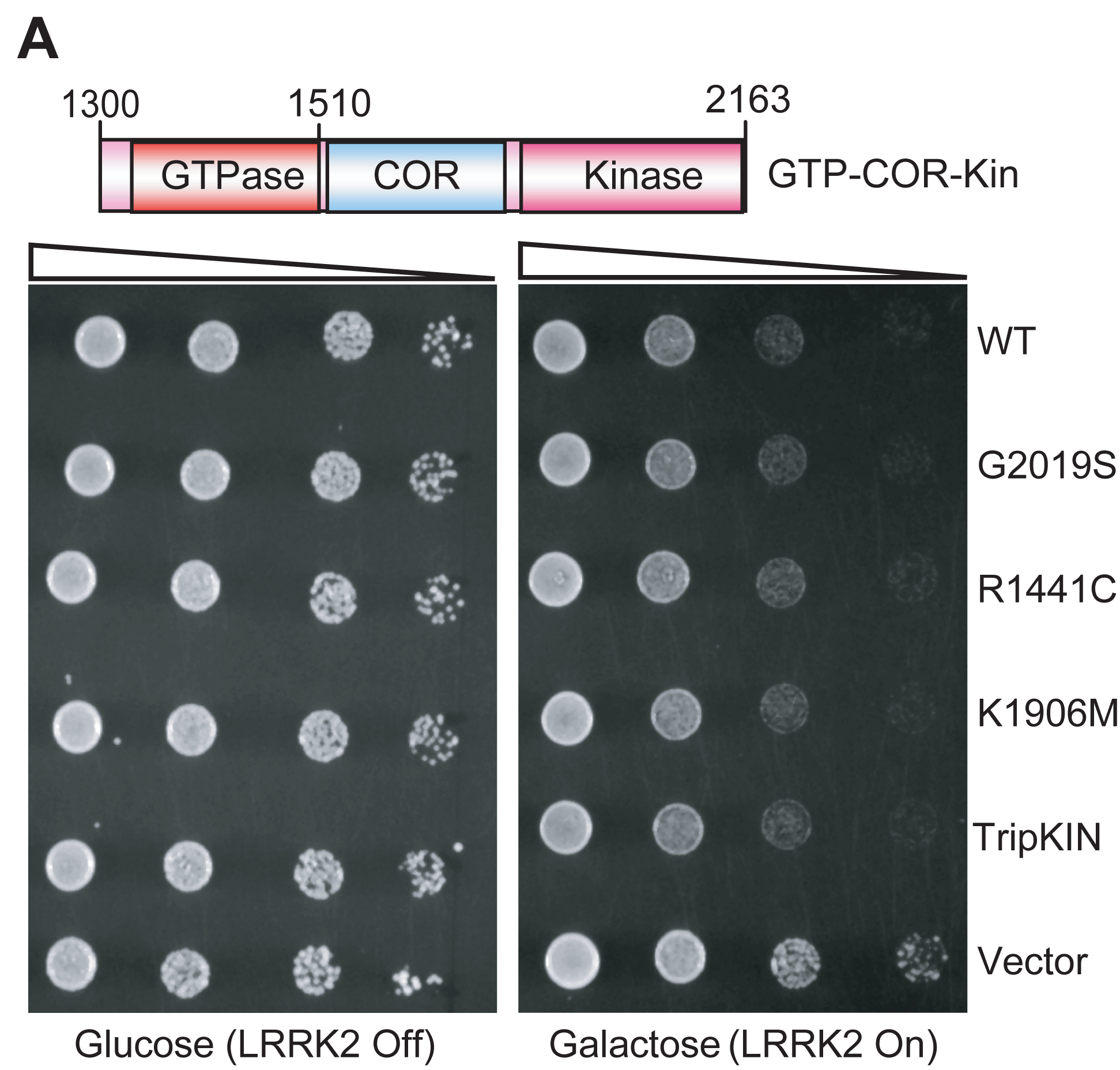

Supplement: Figure S3 — Kinase-modifying mutations fail to influence LRRK2-induced toxicity in yeast. Kinase-modifying mutations were introduced into the kinase domain of the GTP-COR-Kin LRRK2 fragment, including two pathogenic variants that enhance kinase activity (G2019S and R1441C) and two kinase-impaired mutations (K1906M and TripKIN [T2031A/S2032A/T2035A, representing three putative autophosphorylation sites in the kinase activation loop]). Cell viability assay was employed to examine the effects of modulating kinase activity on the viability of yeast cells. Shown are five-fold serial dilutions (from left to right, as indicated by graded open box) starting with equal numbers of cells spotted onto glucose (repressed, off, left panel) or galactose (induced, on, right panel) media. (1.34 MB TIF) [file pgen.1000902.s003.tif]

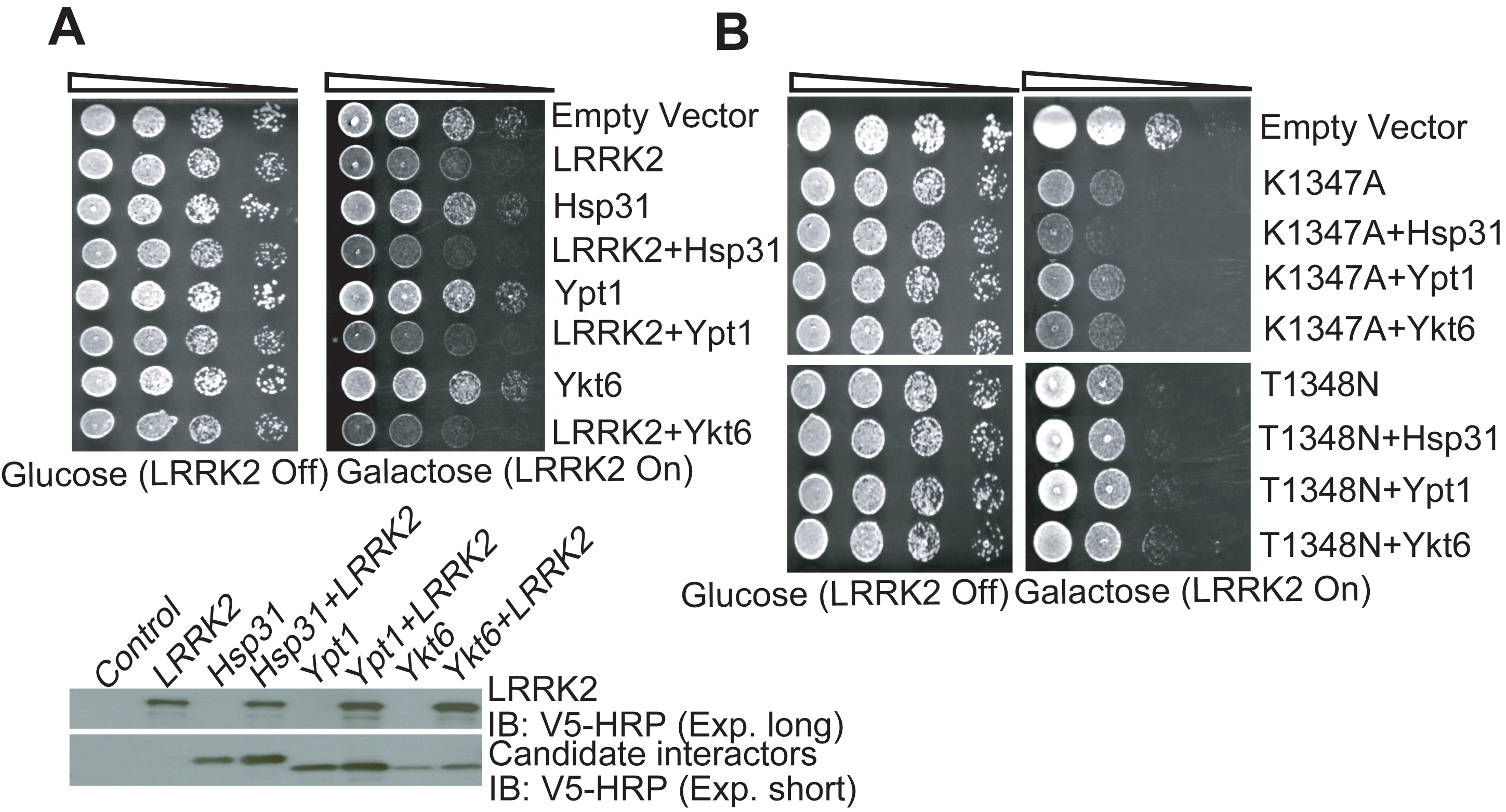

Supplement: Figure S4 — Candidate genetic screen for suppressors of LRRK2-induced toxicity in yeast. (A) Cell viability assay for three candidate genes (HSP31, YPT1, and YKT6) transformed either alone or together with WT LRRK2 (GTP-COR-Kin fragment). Empty vector was used as a control for viability. Shown are five-fold serial dilutions (from left to right, as indicated by graded open box) starting with equal numbers of cells spotted onto glucose (repressed, LRRK2 Off, left panel) or galactose (induced, LRRK2 On, right panel) media. Below, Western blot analysis of total proteins from each yeast transformant following galactose induction probed with anti-V5 antibody to confirm the expression of LRRK2 and each candidate interactor protein. (B) Cell viability assay for three candidate genes co-transformed with LRRK2 GTPase variants, K1347A or T1348N, in the GTP-COR-Kin fragment, compared to single transformation of LRRK2 alone or empty vector as controls. Shown are five-fold serial dilutions (from left to right, as indicated by graded open box) starting with equal numbers of cells spotted onto glucose or galactose media. (3.16 MB TIF) [file pgen.1000902.s004.tif]

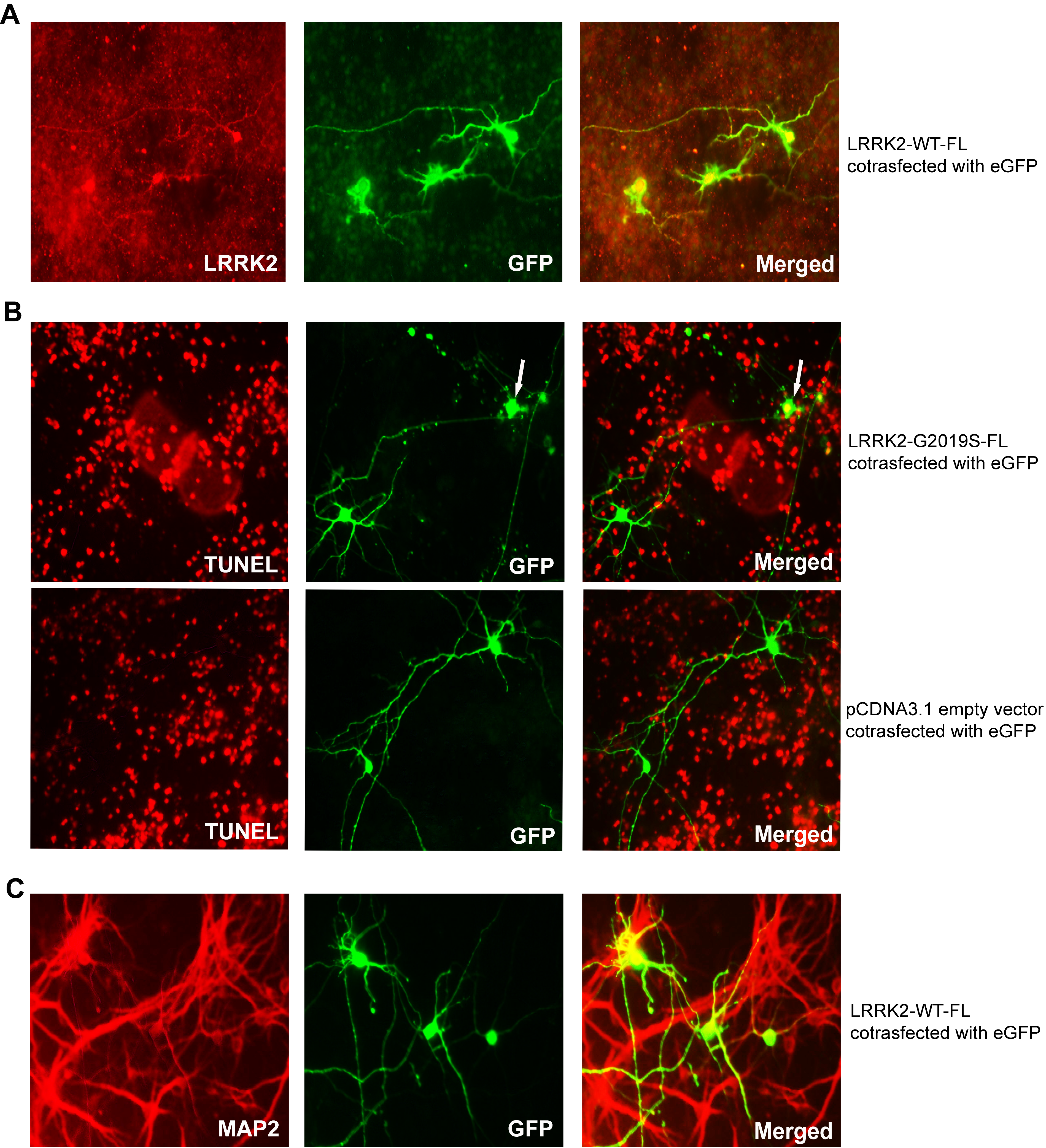

Supplement: Figure S5 — Representative images demonstrating the co-expression of LRRK2 and eGFP, neuronal viability and confirmation of neuronal type in the LRRK2-induced neuronal toxicity assay. (A) LRRK2 is expressed in >95% eGFP-positive neurons when co-transfected with eGFP into primary neurons at a molar ratio of 10∶1. Neurons were co-stained by anti-MYC (LRRK2) and anti-GFP antibodies after 48 hr co-transfection with LRRK2 and eGFP plasmids. (B) Neuronal viability was confirmed by TUNEL staining. Neurons were stained by TUNEL and with anti-GFP antibody after 48 hr co-transfection of LRRK2 and eGFP. The arrow indicates a non-viable eGFP-positive neuron expressing G2019S LRRK2 that also exhibits a TUNEL-positive nucleus. eGFP-positive neurons carrying empty vector or viable neurons expressing G2019S LRRK2 are negative for nuclear TUNEL staining. (C) Confirmation of LRRK2 expression in MAP2-positive cortical neurons. Neurons were co-stained with anti-MAP2 and anti-GFP antibodies after 48 hr co-transfection of LRRK2 and eGFP plasmids at a 10∶1 molar ratio. eGFP-positive neurons expressing WT LRRK2 are positive for the neuronal marker MAP2. (18.51 MB TIF) [file pgen.1000902.s005.tif]
